# Electrophysiological assessment and pharmacological treatment of blast-induced tinnitus

**DOI:** 10.1371/journal.pone.0243903

**Published:** 2021-01-07

**Authors:** Jianzhong Lu, Matthew B. West, Xiaoping Du, Qunfeng Cai, Donald L. Ewert, Weihua Cheng, Don Nakmali, Wei Li, Xiangping Huang, Richard D. Kopke

**Affiliations:** 1 Hough Ear Institute, Oklahoma City, Oklahoma, United States of America; 2 Oklahoma Medical Research Foundation, Oklahoma City, Oklahoma, United States of America; 3 Departments of Physiology and Otolaryngology, University of Oklahoma Health Sciences Center, Oklahoma City, Oklahoma, United States of America; University of Modena and Reggio Emilia, ITALY

## Abstract

Tinnitus, the phantom perception of sound, often occurs as a clinical sequela of auditory traumas. In an effort to develop an objective test and therapeutic approach for tinnitus, the present study was performed in blast-exposed rats and focused on measurements of auditory brainstem responses (ABRs), prepulse inhibition of the acoustic startle response, and presynaptic ribbon densities on cochlear inner hair cells (IHCs). Although the exact mechanism is unknown, the “central gain theory” posits that tinnitus is a perceptual indicator of abnormal increases in the gain (or neural amplification) of the central auditory system to compensate for peripheral loss of sensory input from the cochlea. Our data from vehicle-treated rats supports this rationale; namely, blast-induced cochlear synaptopathy correlated with imbalanced elevations in the ratio of centrally-derived ABR wave V amplitudes to peripherally-derived wave I amplitudes, resulting in behavioral evidence of tinnitus. Logistic regression modeling demonstrated that the ABR wave V/I amplitude ratio served as a reliable metric for objectively identifying tinnitus. Furthermore, histopathological examinations in blast-exposed rats revealed tinnitus-related changes in the expression patterns of key plasticity factors in the central auditory pathway, including chronic loss of Arc/Arg3.1 mobilization. Using a formulation of *N*-acetylcysteine (NAC) and disodium 2,4-disulfophenyl-*N*-tert-butylnitrone (HPN-07) as a therapeutic for addressing blast-induced neurodegeneration, we measured a significant treatment effect on preservation or restoration of IHC ribbon synapses, normalization of ABR wave V/I amplitude ratios, and reduced behavioral evidence of tinnitus in blast-exposed rats, all of which accorded with mitigated histopathological evidence of tinnitus-related neuropathy and maladaptive neuroplasticity.

## Introduction

Tinnitus has been linked to common sequelae of acoustic overexposures [1–3]. Tinnitus occurs in 14–20% of the population and is one of the most prevalent service-related disabilities in the U.S. military [4, 5]. Despite its high prevalence and potentially distressing impact on quality of life, there is currently neither a pharmacologic therapy nor an objective clinical test for tinnitus.

The study of the underlying mechanisms that drive tinnitus has increased exponentially over the past several decades, and the prevailing view has shifted to a belief that tinnitus, even when triggered by peripheral cochlear damage, originates within the central auditory system [6–12]. This unifying “central gain” model proposes that tinnitus results from compensatory increases in gain (or sensitivity) at virtually all levels in the central auditory system due to loss of cochlear sensory input [10, 13, 14].

As such, peripheral auditory damage represents a primary risk factor for the generation of tinnitus, where inner hair cell (IHC) deafferentation, rather than outer hair cell (OHC) loss, is posited to play a key role in driving the disorder [15–19]. Animal studies have revealed that acoustic traumas, causing only transient threshold elevations can, nonetheless, cause immediate and permanent loss of cochlear ribbon synapses, which is consistent with clinical observations in which people with normal audiograms also report tinnitus [20–24].

However, the identification of objective electrophysiological measurements that effectively define and capture evidence of this neurological phenomenon have proven difficult to develop. Auditory brainstem responses (ABRs) in tinnitus subjects have been extensively investigated with the hopes of finding possible abnormalities related to the putative neuropathology, however a consistent objective signature has proven challenging to resolve [25–28]. Previous work has shown that (1) reduced suprathreshold amplitudes of ABR wave I represent cochlear neuropathy [20, 29], and (2) following peripheral deafferentation, enhanced (or sustained) amplitudes of ABR wave V, which is generated in the lateral lemniscus and inferior colliculus, are indicative of central hyperactivity [10, 29, 30]. Based on central gain control in tinnitus, the resultant ratiometric elevation in ABR wave V/I amplitudes has, thus, been proffered as a potential objective signature for diagnosing tinnitus.

As prior work has revealed that IHC deafferentation represents a logical catalyst for tinnitus, potential therapeutic approaches for their treatment would seemingly be best addressed by preserving or regenerating these peripheral synaptic connections. Oxidative stress is considered to be one of the main causes of neural damage after acoustic injury. Previous studies have shown that peripheral nerve injury induces the production of reactive oxygen species and nitric oxide in axotomized neurons [31]. In addition, excessive release of glutamate from these afferent synapses has long been considered to be a major cause of excitotoxic ribbon synapse and neurite loss [32]. Thus, antioxidants and spin trap agents that remove excess free radicals and protect against progressive oxidative injury and excitotoxicity are currently being advanced into potential neurotherapeutics for preservation of neurons and their fragile neuritic processes and synaptic connections [33, 34]. Our prior studies with *N*-acetylcysteine (NAC) combined with 2,4-disulfophenyl-*N*-tert-butylnitrone (HPN-07) have demonstrated that the two drugs act synergistically to decrease oxidative stress, neuroinflammation, apoptosis, ischemia-reperfusion injury, and neurodegeneration throughout the auditory pathway [35–43]. Based on the pervasive mitigative effects of NAC/HPN-07 in addressing acute and progressive histopathological sequelae in response to a diverse array of acoustic traumas, we hypothesized that the drug combination might also demonstrate efficacy against peripheral and central manifestations of tinnitus-related neuropathy.

The goals of the present study were five-fold: (1) to survey central auditory structures of mild blast-exposed rats for histological evidence of chronic tinnitus-related neuropathy/neuroplasticity; (2) to validate the blast model for the generation of a chronic tinnitus percept; (3) to reveal the peripheral factors that represent likely triggers for tinnitus; (4) to establish a regression equation for predicting tinnitus; and (5) to test therapeutic effects of NAC combined with HPN-07 on addressing behavioral, physiological, and histopathological evidence of tinnitus.

Here, we demonstrate that, in animals with behavioral evidence of tinnitus, deafferentation of cochlear nerve fibers triggers compensatory neural plasticity in the central auditory pathway, resulting in the development of tinnitus. Our results revealed that NAC/HPN-07 therapy reduced the risk of tinnitus by approximately 50%, wherein cochlear ribbon synapse preservation or repair and normalization of the expression patterns of homeostatic gating factors in the central auditory pathway were apparently involved. Our regression analyses suggest that ABR wave-V/I amplitude ratios can serve as an objective and reliable tinnitus metric and can provide evidence of treatment-mediated normalization of aberrant central plasticity changes.

## Materials and methods

### Animals

All procedures regarding the use and handling of animals were approved by the Oklahoma Medical Research Foundation Institutional Animal Care and Use Committee. Eighty Long–Evans male rats, aged 1–3 months, were used in histologic, diagnostic, and therapeutic studies on blast-induced pathology and tinnitus. Rats weighing 250–300 g were obtained from Harlan Laboratories and were housed in the Oklahoma Medical Research Foundation (OMRF) animal care facility. All animals were evaluated by the attending veterinarian upon arrival and were monitored daily by a trained staff of laboratory animal technicians. Animals were maintained on a normal day/night cycle at 21°C with free access to food and water, where following the usual routine, they were provided a 1-week or more acclimation period prior to experimentation. For the electrophysiological, behavioral, and ribbon synapse studies, rats were divided into three groups of twenty: (1) unexposed control group; (2) nontreatment group, in which animals underwent blast exposure and received vehicle treatment with saline; and (3) treatment group, in which animals were subjected to the same blast exposure and received therapeutic treatment with NAC/HPN-07 dissolved in saline solution. For the spiral ganglion neuron and brain biomarkers studies, an independent group of twenty rats were divided into the same experimental cohorts and were subjected to blast and subsequent vehicle or NAC/HPN-07 treatment as described above. All procedures regarding the use and handling of animals were approved by the OMRF Institutional Animal Care and Use Committee.

### Blast exposure

Various blast simulators used in laboratory settings have been developed to generate blast overpressure waves that closely resemble a classic Friedlander wave, with a positive phase and negative phase above and below atmospheric pressure, respectively [44]. Here, our simulating device was a compressed nitrogen gas-driven blast simulator engineered to be mounted vertically within the confines of a sound booth through modification of a previously-described blast simulator [41]. The internal geometry of the shock tube (circular vinyl tube with 48″ in length and 8″ in inner diameter) conformed to the specifications of the conical blast simulator described by Ritzel *et*. *al*. (2011), whereby the cross-section of the interior of the upper end of the tube increased proportionately to the square of the axial distance [45]. The shock tube consisted of two essential sections of substantially different pressures—namely, the driver (breech) and the driven that were separated by a polycarbonate membrane (1.0 mm thick, McMaster-Carr) that was secured between the compression chamber and the expansion chamber of the shock tube. A pneumatic piston pushed the fixed volume breech against the film sheet, forming the airtight chamber. Compressed nitrogen gas filled the breech until the increase in pressure inside exceeded the strength of the film to generate a membrane burst. As a result, the generated overpressure propagated along the driven section, causing a shock wave at the leading edge followed by a decay in the pressure profile, which approximated an ideal blast wave.

Following anesthesia with 90 mg/kg ketamine and 9 mg/kg xylazine, animals were placed inside the shock tube in a rostro-cephalic orientation towards the blast source and were exposed to a single blast wave of 10 psi peak overpressure. Only the head of each rat was subjected to blast exposure, whereas its body was protected from blast injury via a small circular polyvinylchloride holding tube (6″ long with inner diameter of 2″), which was mounted on rails on a metal frame that allowed for precise positioning of a rat along the X-, Y- and Z-axis to ensure that each animal was positioned at a point where the time-pressure profile of the blast wave formed a 10 psi shock front that corresponded to a Friedlander wave. Each 360-400g rat fit snuggly within the holding tube, with only its head exposed to the base of the pinna. Blast wave profiles were verified to maintain consistent exposure pressures between subjects. The overpressures were measured inside of the shock tube by means of a high-frequency piezo-electronic pressure transducer (PCB Piezotronics, Inc., Depew, NY), while the data acquisition and analyses were performed with a PicoScope 3000 oscilloscope (PCB Piezotronics, Inc.).

### Drug administration

NAC was purchased as a 20% solution from Hospira, Inc. HPN-07 was synthesized and provided at greater than 98.5% purity by APAC Pharmaceuticals, LLC. A mixture of both drugs was prepared in saline to reach a final concentration of 60 mg/mL for each drug. Drug therapy was administered intraperitoneally (i.p.) at 5 mL/kg, starting at 1h after blast exposure and then continuing twice daily for the following two days. Animals in the vehicle control group (which we refer to as the vehicle group in the present study) were injected i.p. with the same volumetric ratio of saline (5 mL/kg) as the active drug formulation, according to the same schedule as the treatment group.

### Histology: Biomarker evaluations

Animals used for brain and cochlear sectioning and subsequent immunohistological evaluations in each experimental group (6–8 rats/group) were euthanized and intracardially-perfused with saline followed by 4% paraformaldehyde in 0.1 M phosphate-buffered saline (PBS, pH 7.2) at nine weeks post-blast. Brains, brainstems and cochleae were removed and post-fixed in the same fixative (one week for brain tissues and overnight for cochleae), washed with PBS, and then stored in PBS at 4°C. The brain and brainstem from each animal were cryoprotected in 30% sucrose in PBS at 4°C until the tissue settled to the bottom of the container, at which time they were embedded in Tissue-Tek (Sakura Finetek USA Inc. Torrance, CA) and serially sectioned in a coronal plane with a Thermo Cryotome (Thermo Fisher Scientific, Inc. Waltham, MA) at 20 μm. One section out of every ten from each brain and brainstem was mounted onto a gelatin pre-coated slide (total of 10 slides for each brainstem and 20 slides for each brain); the distance between two adjacent sections on each slide was about 200 μm. Sections were blocked in 1% bovine serum albumin (BSA, fraction V) and either 1% normal horse serum or 1% normal goat serum in PBS, and permeabilized in 0.2% Triton X-100 in PBS (PBS/T). Blocked and permeabilized sections were then incubated with either rabbit anti-Arc (1:100, abcam, Cambridge, MA, catalog# ab23382), rabbit anti-GABA_A_R-α1 (1:500, EMD Millipore, Billerica, MA, catalog# 06–868), or mouse anti-GluR2 (1:100, EMD Millipore, Billerica, MA, catalog# MAB 397) overnight at room temperature. After washing with PBS/T, either biotinylated goat anti-rabbit IgG or horse anti-mouse IgG (1:200, Vector Laboratories, Inc. Burlingame, CA) was applied to the slides for one hour at room temperature, and Vectastain ABC and DAB kits (Vector Laboratories, Inc. Burlingame, CA) were used for the immunolabeling visualization. Immuno-positive cells exhibited a brown reaction product at the sites of the target epitopes. Methyl green was used for nuclear counter-staining. Negative controls were prepared by omitting the primary antibodies. For dual immunofluorescence evaluations of GABA_A_R-α1 and GAD67, sections were blocked as described above and then incubated with mouse anti-GAD67 antibody (1:100, EMD Millipore, Billerica, MA, catalog# MAB5406) and rabbit anti-GABA_A_R-α1 (1:500) overnight at room temperature. After washing with PBS, the sections were incubated with appropriate Alexa Fluor^®^ 488 and 568 secondary antibodies (1:1000, Life Technologies, Co., Grand Island, NY) for two hours at room temperature followed by DAPI nuclear labeling and mounting in anti-fade medium. Images were collected with a Zeiss LSM-710 confocal microscope. Antigen retrieval (10 mM sodium citrate, 0.05% Tween 20, pH 6.0, 58°C overnight) was employed for GluR2 immunostaining, as well as in GAD67 and GABA_A_R-α1 dual immunolabeling.

For quantitative evaluations, images were collected with a BX51 Olympus microscope. In the brain, dorsal cochlear nucleus (DCN) images were collected from the medial third (*medial*), the middle third (*middle*) and the lateral third (*lateral*) sections. In the inferior colliculus (IC), images were collected from the central nucleus of the IC. In the auditory cortex (AC), images were collected from all layers (two images to cover all layers on one section). A modified two-dimensional quantification method was employed to count immuno-positive cells in these nuclei or regions [38, 46]. The total number of immune-positive cells within each image was quantified using ImageJ software (National Institutes of Health) by a technician who was unaware of the identity of the samples on each slide. Only dark brown-stained cells were counted. The density of each biomarker-positive cells (number of positive cells/mm^2^) was calculated and statistically analyzed.

For VR1 immunostaining in the spiral ganglion (SG), fixed cochleae were washed with PBS and then decalcified for two weeks in 10% EDTA with solution changes two times each week. Cochleae were then dehydrated, embedded in paraffin, and sectioned in a paramodiolar plane at a thickness of 6 μm. Every 10th section was mounted on a slide (total of 10 slides per cochlea), and the mounted sections were processed for immunohistochemical analyses (as detailed below). Cochlear sections were de-paraffinized in xylene and re-hydrated in serial concentrations of ethanol and distilled water. The sections were blocked with 1% BSA and 1% normal goat serum in PBS for 1 hour and then incubated with rabbit anti-VR1 antibody (1:1000, EMD Millipore, Billerica, MA, catalog# AB5370) overnight at room temperature followed by visualization processing as detailed above. Images were collected from the spiral ganglion in the basal and middle turns of all sections on each slide. The number of VR1-positive neurons was quantified using ImageJ software. The percentage of VR1-positive neurons in the SG (positive stained/total number of neurons x 100%) was calculated and statistically analyzed.

### Physiology: Auditory brainstem responses (ABRs)

ABRs were measured in a sound booth (Industrial Acoustics Company) in each individual ear of blast-exposed rats and unexposed naïve control rats at four time points—i.e., prior to blast exposure and at 24 h, 4 wk and 8 wk after exposure. For testing, animals were anesthetized with ketamine (90 mg/kg) and xylazine (9 mg/kg) via intraperitoneal injection, while basal body temperature was maintained with a servo heating pad. Stimulus generation and data acquisition were accomplished with a RX6 workstation (Tucker-Davis Technologies, Inc., or abbreviated as TDT), running TDT BioSigRP software. Acoustic stimuli were tone bursts of alternating polarity, with 5-ms plateau and 0.5-ms cos^2^ rise-fall envelope at frequencies of 4, 8, 16, and 24 kHz. The stimuli were delivered to the ear in free-field mode by a TDT MF1 speaker placed 10 cm lateral to the animal’s pinna. The MF1 was driven by a TDT stereo power amplifier. Electrical brainstem responses to tone bursts were recorded using sub-dermal needle electrodes located at the vertex (active), the ipsilateral mastoid (reference) and the contralateral mastoid (ground), where the responses were amplified 5,000 × before digitization, band-pass filtered from 0.3 to 3 kHz, and averaged across 512 repetitions at a rate of 21/s. Sound intensities were varied in 5-dB steps up and down to identify ABR threshold. The threshold was defined as the lowest dB stimulus level that yielded a repeatable, clearly discernible waveform by visual analysis of stacked waveforms from highest to lowest SPL. Amplitudes of waves I and V of the ABR in response to a stimulus level of 80 dB SPL were measured for analysis of the wave-V/I amplitude ratio. The wave amplitude was defined as the height of a peak in microvolts from the top of the peak to the lowest point of the following negative trough, which is known as the peak-to-peak amplitude.

### Behavioral testing: Prepulse inhibition (PPI) of the acoustic startle response

PPI tests were conducted in blast-exposed rats and in unexposed, age- and sex-matched naïve control rats before exposure and at 4- and 8-weeks after exposure. The PPI detection method was adapted from Turner *et al*. (2006) and was based upon the ability of the acoustic startle response to be considerably reduced when preceded by a silent gap in a constant acoustic background [47]. The rationale for assessing tinnitus is that the phantom percept would fill in the silent gap embedded in the background sound, thus resulting in gap PPI deficits. By means of the StartleMonitor/AuxAmp II system (Kinder Scientific, LLC), behavioral testing was performed inside of a sound-attenuating box with two loudspeakers mounted in its ceiling and with a piezoelectric transducer platform attached to its floor. The two speakers were arrayed front-to-back to present the startle pulse via the front one and the background noise/acoustic prepulse via the back one. The transducer converted the vertical startle movement of the platform into a voltage signal. The data-sampling window was 500 ms, where the startle analysis window was set for a 100-ms area following startle-pulse onset. The startle pulse was a broadband noise (BBN) burst of 50 ms in duration with 0-ms rise-fall times. The intensity was set at 107 dB SPL where the input/output function of acoustic startle could reach the plateau of maximum startle response. Here, there were two modalities of prepulses in the PPI tests, namely the acoustic prepulse (a narrow-band noise burst) and the gap prepulse (a silent interval embedded in an otherwise continuous background noise known as the gap carrier). The prepulse envelope was 50 ms long with 1.0-ms ramps, occurring 100 ms before the startle pulse, where the acoustic prepulse and the gap carrier were filtered noise (1/3-octave bandpass, 48 dB/octave roll-off) centered at frequencies of 9.3, 16, 20 and 24 kHz, each at an intensity of 60 dB SPL. A trial with a prepulse-pulse and a trial with a startle pulse alone were presented in a pseudorandomized order to perform PPI testing, where the startle pulse alone trial for gap PPI was identical to the one for acoustical PPI, except for the presence of continuous background noise. The behavioral testing protocol was divided into an acclimation period (2 min), a block I (short-term habituation) and a block II (PPI tests).

During the two-minute acclimation phase, animals adapted to the test environment (e.g. the animal holder and startle box). In block I, three startle pulse alone trials were run in quiet to habituate the startle response to a more stable baseline. No data were collected in this block. In block II, we used four different frequencies (9.3, 16, 20 and 24 kHz) to match tinnitus pitch. Thus, there were four different prepulse-pulse trials, each paired with a startle pulse alone trial to be displayed under each of the two prepulse conditions (i.e., the acoustic and gap versions of the PPI paradigm). A total of eight pairs of trials were pseudorandomized and presented 12 times, each throughout block II, which had 8×2×12 = 192 trials. The twelve data traces per trial type for the same pair were averaged, and the resulting value for the prepulse-pulse trials was divided by the startle pulse alone value. This quotient was the amount of PPI for each subject for each given frequency, where a value of 1 means no effect of the prepulse, a value of 0 means complete prepulse inhibition of the startle. In some PPI trials, however, absolute response amplitudes differed considerably between presentations of the same trial. As such, we applied Grubb’s test to determine if the most extreme value in a set of trials of the same type was an outlier by analysis of its Z-score (where $z\;=\;\frac{|extreme\;value-mean|\;}{SD}$). When the Z-score of a given trial was > 2.41 (i.e. the G critical value for a data set of 12 numbers), we rejected this trial as an outlier. We performed Grubb’s test only once for each trial set. Also, for some animals, acoustic startle responses appeared to be too small to be appropriate for PPI measurements. Considering this issue, activities from the startle pulse alone trials (that were presented without background noise) were analyzed by using the first and second recording windows (that were set for a 100-ms area before and after startle-plus onset, respectively). Animals were excluded from PPI analysis if they met the following criterion: waveform peaks in the second window were < 3 standard deviations above the highest activity levels that occurred in the first window. Therefore, within each window, for each animal, activities were averaged across all startle pulse alone trials (48 trials) that were extracted from acoustic PPI testing in bock II. In blocks I and II, inter-trial intervals varied randomly between 5 and 11 sec. This variable interval was aimed at preventing the experimental animal from predicting the time point of the prepulse, since attention to the prepulse may augment its efficacy in suppressing startle responses. Also, as described by Valsamis and Schmid (2011), both startle attenuations (due to long-term habituation) and PPI improvements (due to PPI learning) are two important processes affecting the behavior [48]. To minimize their impact on the animal, we performed the entire testing protocol on five consecutive days. The actual data collection took place during the last two days. The results of the two-day tests were averaged for each animal.

Tinnitus determination was based upon the method of Sametsky *et al*. (2015) in the use of tinnitus index score as an assay for tinnitus [49]. Tinnitus index scores were calculated by subtracting the acoustic PPI from the gap PPI. Higher scores are indicative of tinnitus-like deficits in processing silent gap cues. When compared to the commonly used gap PPI for determining tinnitus, the index score helps to control for some potential confounding factors (e.g. startle reflex amplitude differences, hearing loss, sensory gating failures) in the gap ratio as a standalone metric. The extraneous influence should be present in both the gap-PPI and acoustic PPI conditions.

### Histology: Synaptic ribbon counts

Cochlear tissue collection and ribbon synapse counting have been described previously [40]. In brief, animals were decapitated under deep anesthesia after the terminal ABR and PPI tests (approximately nine-weeks post-blast). Cochleae were quickly removed from the temporal bones and placed in cold PBS. The round and oval windows were opened, and a hole was made at the apical portion. Cochleae were perfused with 4% formaldehyde solution in PBS and post-fixed for an additional 10 min at 4°C. Cochleae were then dissected in PBS and blocked with 1% Triton X-100 and 5% normal horse serum PBS for 1 hour. Cochlear tissues were immunolabeled with mouse anti-C-terminal binding protein antibody (CtBP2, BD Transduction Laboratories, catalog # 612044, 1:200) for 20 h at 37°C. The tissues were incubated with Alexa Fluor568 goat anti-mouse antibody (1:1000, Life Technologies, Co.) for 1h at 37°C. The tissues were counterstained with DAPI (4’,6-diamidino-2-phenylindole) for 10 min at room temperature to label nuclei and then mounted on slides with anti-fade medium (ProLong Gold, Thermo Fisher Scientific Inc, Rockford, IL). The whole cochlea was photographed with an epifluorescence microscope. Cochlear length was measured, and a frequency map was created using a custom ImageJ plug-in (http://www.masseyeandear.org/research/ent/eaton-peabody/epl-histologyresources/). Confocal z-stacks at the 2, 4, 8, 16, 32 and 48 kHz regions were obtained. Images were acquired using a Zeiss LSM-710 confocal microscope (Carl Zeiss Microimaging, LLC), in a 1024×1024-pixel frame with z steps of 0.5 μm. From an endolymphatic surface view of the organ of Corti, each stack contained ribbon synapses of six to nine IHCs. Image stacks were processed using Amira 3D software (FEI). All quantitative analyses were performed with raw image stacks. The presynaptic ribbons (red channel) were identified by segmentation, then quantified and tracked in the z-dimension to avoid superpositional ambiguity or overestimations in each stack. Individual ribbons were isolated, counted, and expressed as synaptic ribbons per IHC in the stack.

### Statistical analyses

For quantitative biomarker analyses, one-way ANOVA (SPSS 14.0 for windows) and a *post hoc* test (Tukey) were used to determine if there were statistically-significant differences among groups and between each of two groups. *P* values of less than 0.05 were regarded as statistically-significant.

For mean tinnitus index scores, a confidence interval was constructed at a confidence level of 95% for each test frequency using data from the control group. The interval that we can be 95% certain contains the true mean index score in the normal population was applied as a diagnostic criterion for tinnitus. That is, blast-exposed subjects with scores falling above the 95% confidence interval of the companion control group were considered to have tinnitus at that given test frequency. Fisher’s exact test was used to evaluate whether the treatment and non-treatment groups would differ in proportion developing tinnitus. In most cases, two-factor ANOVAs with “frequency” × “group” (or “time”) were performed on datasets of our observed response variables (e.g. amplitude and amplitude ratio of ABRs, PPI of startle, synaptic ribbon count of IHCs) to find the differences between the control and exposure groups. Here, “group” was commonly referred to as a between-subjects factor, while “frequency” was always treated as a within-subjects factor (also known as a repeated-measures factor) since our experimental design was so that we took multiple measures across frequencies per subject. When investigating changes in mean values over two or more time points, which were measured in the same exposure group, we would treat “time” as a within-subjects factor as well to partition out the variability due to the individual differences between subjects. This has the effect of increasing the value of the *F*-statistic and leading to a more powerful ANOVA test. When performing a two-factor ANOVA with repeated measures in either one factor (a mixed ANOVA) or both factors (a two-way repeated measures ANOVA), we herein assumed that sphericity could hold, which is the requirement for ANOVA with matched samples. Note that if there are only two levels of repeated measures then sphericity is automatically satisfied. In this study, the factor “time” that was repeated measures had only two levels (4- and 8-weeks post-exposure), then there is no reason to be concerned about violations of sphericity. If a significant interaction of “frequency” with “group” (or “time”) was found in ANOVA, we examined the simple effect of “group” (or “time”) at each specified value of “frequency”. Simple effects were assessed for significance using the Holm-Bonferroni method. Here, binary logistic regression was employed to achieve two goals: (1) to determine whether there was a relationship between the amplitude ratio V/I of ABRs and the presence/absence of tinnitus, in hopes of understanding more about the mechanism of tinnitus; and (2) to find an equation that would predict the probability of tinnitus with a given ratio V/I, to help guide development of an objective measure for tinnitus. The fit of logistic regression model was tested using the following measures: likelihood ratio test, Wald test, Hosmer-Lemeshow test, classification table, ROC curve and pseudo R-squareds. Also, loess (“local regression”) analysis was used for fitting a smooth curve between the ABR wave-V/I ratio and either of ABR threshold shifts and cochlear synapse counts to reveal whether hearing loss and/or cochlear synaptopathy would affect the ratio of ABR wave V to wave I. Statistical analysis was performed using Real Statistics Using Excel (http://www.real-statistics.com/) for logistic regression, R version 3.4.3 (http://www.r-project.org/) for loess curve fitting, and GraphPad Prism 4 (GraphPad Software, Inc.) for ANOVA, respectively.

## Results

### Chronic blast-induced histopathological changes in central and peripheral auditory pathways

In previous studies of rats exposed to mild blast injuries, chronic neuronal spontaneous hyperactivity in central auditory nuclei has been reported as a conjunctive sequela associated with peripheral hearing loss [50–52]. This blast-induced hyperactivity has, in turn, been shown to closely correlate with behavioral evidence of chronic tinnitus, under conditions in which a low to moderate degree of permanent ABR threshold shift was evoked by the acoustic trauma. In our prior studies with an open field blast model, we demonstrated that a combinatorial treatment of NAC/HPN-07, administered shortly after blast, was capable of addressing both peripheral and central manifestations of neuropathy but had not extended these studies to examine whether this treatment was capable of addressing chronic neuroplastic reorganization in the central auditory pathway and potential tinnitus-related dysfunction [39–41]. Thus, the current study was designed to determine whether NAC/HPN-07 treatment can (1) normalize chronic, tinnitus-related molecular responses in the central and peripheral auditory pathways; (2) therapeutically-mitigate the development of a chronic tinnitus percept; and (3) provide insights into key mechanistic triggers for the generation of tinnitus after blast exposure.

To examine whether we could detect objective molecular evidence of chronic blast-induced neuroplastic changes and whether these putative response patterns were addressable by NAC/HPN-07 treatment, we exposed rats to a single shock tube blast of 10 psi (~190 dB peak SPL), which consistently induced minor to moderate permanent ABR threshold shifts in untreated controls of 8.7±1.1, 8.0±1.4, 15.8±1.4, and 42.0±2.7 dB at 4, 8, 16, and 24 kHz, respectively, at eight weeks post-blast in our electrophysiological evaluation studies. Treatment with either vehicle (saline) or NAC/HPN-07 was initiated at one-hour post-injury, and twice-daily treatments were repeated on two consecutive days thereafter. Animals were then allowed to recover for nine weeks prior to harvesting tissues for histopathological examination of chronic blast-induced changes.

**Fig 1:**
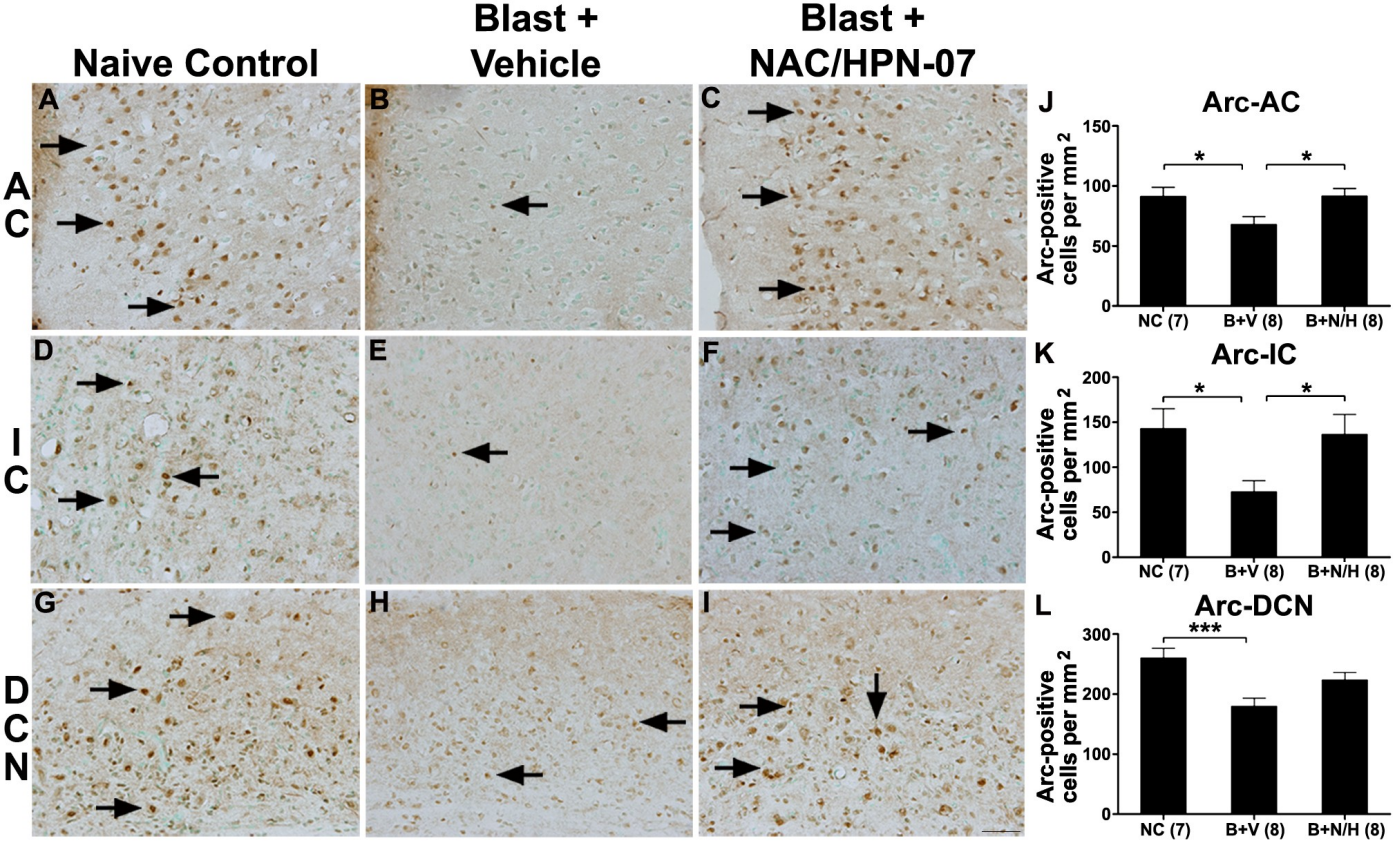
NAC/HPN-07 treatment reduced the blast-induced down-regulation of Arc in the central auditory system. Examples of Arc immunohistochemical staining in the AC, the IC and the DCN of the naive control group (*arrows* in A, D and G), the vehicle-treated, blast- exposed group (*arrows* in B, E and H) and the blast exposed, therapeutic-treated group (*arrows* in C, F and I). The number of Arc-positive neurons in the AC, the IC and the DCN was counted and statistical analyzed (J, K L). Statistical significance was determined by ordinary one-way ANOVA (Tukey correction for multiple comparisons). The number of Arc-positive neurons was significantly decreased in the AC (J, * *p* < 0.05), the IC (K, * *p* < 0.05) and the DCN (*** *p* < 0.001) in vehicle-treated rats exposed to blast (*B+P*) compared to naive controls (*NC*). Significant recovery of Arc-positive neurons was observed in the AC and the IC in rats exposed to blast and then treated with NAC/HPN-07 (*B+T*) compared to vehicle-treated rats exposed to blast (*, *p* < 0.05). No significant recovery was observed in the DCN after NAC/HPN-07 treatment (L, *p* > 0.05), although these animals showed a trend of therapeutic recovery in this auditory center. Numbers in parentheses represent the number of animals evaluated in each group. The scale bar in I = 50 μm, and applies to A-I.

Brain tissues from central auditory nuclei of the blast-exposed rats in each cohort were immunolabeled with antibodies directed against activity-regulated cytoskeleton-associated protein (Arc, also known as Arg3.1), gamma-aminobutyric acid-type A receptor alpha 1 (GABA_A_R-α1), and glutamate receptor 2 (GluR2). Arc is a neuroplasticity protein that regulates synaptic strength by enhancing the endocytic trafficking rate of AMPA receptors and has been widely used as a tinnitus biomarker in the brain [26, 53–56]. Seminal work by Singer and colleagues demonstrated that a persistent failure to mobilize Arc in the central auditory pathway after an acoustic trauma differentially predisposed animals for the development of a chronic tinnitus percept [26]. To examine the effects of our shock tube blast exposure model on Arc expression patterns in the central auditory pathway, Arc immunostaining was conducted among neurons in the auditory cortex (AC), the inferior colliculus (IC), and the dorsal cochlear nucleus (DCN), which were previously shown to be key nuclei for blast-induced neuroplasticity in the central auditory pathway [50–52]. In naïve control animals, numerous Arc-positive neurons were observed in the AC, IC and DCN (*arrows* in Fig 1A, 1D and 1G). In the AC, the majority of immunoreactive neurons were located in the superficial layers (*arrows* in Fig 1A). Few Arc-positive neurons were observed in the central nucleus of the IC (*arrows* in Fig 1D). In the DCN, the majority of Arc-positive neurons were located in the fusiform soma layer (FSL, *arrows* in Fig 1G).

In vehicle-treated, blast-exposed rats, chronic reductions in Arc immunoreactivity were observed in the AC, IC and DCN at nine weeks post-blast relative to naïve controls (*arrows* in Fig 1B, 1E and 1H). Quantification and statistical evaluations revealed that the number of Arc-positive neurons in vehicle-treated, blast-exposed animals were, indeed, significantly reduced in the AC, IC and DCN compared to naive control rats (*NC vs B*, ** *p* < 0.05 or 0.001, one-way ANOVA with Tukey post-test, Fig 1J, 1K and 1L). In this sample set, 75% of vehicle-treated rats presented with statistically-significant reductions in the number of Arc-positive neurons in the DCN and IC, while 62.5% presented with significant reductions in the AC. The pervasive changes in Arc immunoreactivity, particularly in the DCN and IC, observed in blast-exposed rats is suggestive of maladaptive remodeling in these auditory centers in response to blast trauma. Consistent with this hypothesis, we also observed persistent upregulation of the plasticity marker, GAP-43, in the DCN and IC among animals in this cohort (S1 Fig).

In blast-exposed rats treated with NAC/HPN-07, the density of Arc immunostaining in these same brain regions was grossly indistinguishable from naïve controls (*arrows* in Fig 1C, 1F and 1I). Quantitative evaluations confirmed significant, treatment-specific recovery of the number of Arc-positive neurons in the AC and IC of blast-exposed animals treated with NAC/HPN-07 compared to vehicle-treated controls (* all *p* < 0.05), such that there was no significant difference between the naïve controls and the NAC/HPN-07 treatment groups in the AC and the IC (*p* > 0.05). Although there was a clear recovery trend for Arc immunoreactivity in the DCN in response to NAC/HPN-07 treatment, statistical significance could not be assigned relative to the vehicle-treated group in this auditory center (Fig 1L, *p* > 0.05, Table 1).

**Table 1:** Summary of blast-induced biomarker changes in the auditory system.

Biomarker	DCN		IC		AC		SG	
	P	TE	P	TE	P	TE	P	TE
**ARC**	**↓**	**No**	**↓**	**Yes**	**↓**	**Yes**		
**GluR2**	**↑**	**Yes**	**-**	**No**	**-**	**No**		
**GABAA-Rα1**	**↑**	**Yes**	**-**	**No**	**↑**	**Yes**		
**GAP43**	**↑**	**Yes**	**↑**	**Yes**	**↑**	**Yes**		
**VR1**							**↑**	**Yes**

*Abbreviations*: P, vehicle-treated rats exposed to blast; TE, therapeutic effects among NAC/HPN-07-treated, blast-exposed rats; DCN, dorsal cochlear nucleus; IC, inferior colliculus; AC, auditory cortex; SG, spiral ganglion.

“↑”, significantly more immunopositive cells compared to naive controls; “↓”, significantly fewer immunopositive cells compared to naive controls; “-“, no change compared to naive controls. “Yes” and “No” refer to positive or negative therapeutic effects relative to vehicle-treated controls.

**Fig 2:**
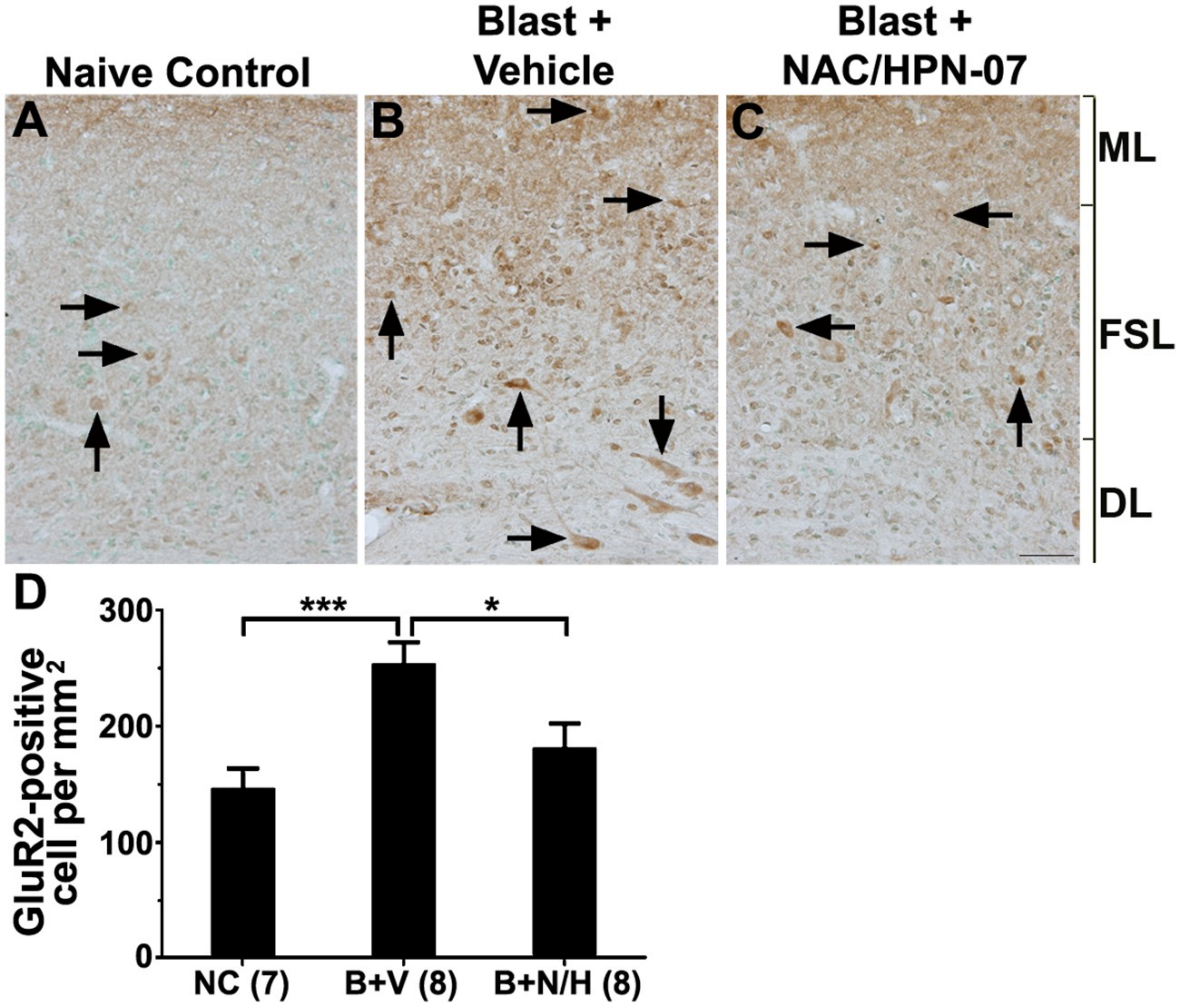
NAC/HPN-07 treatment reduced chronic accumulation of GluR2 in the DCN of blast-exposed rats. Examples of GluR2 immunostaining in the DCN from the naive control group (*arrows* in A), the vehicle-treated, blast-exposed group (*arrows* in B), and the blast-exposed, therapeutic-treated group (*arrows* in C). GluR2 positive-neurons were largely restricted to the fusiform soma layer (*FSL*) in the naive control group (*arrows* in A) and in blast-exposed rats treated with NAC/HPN-07 (*arrows* in C). In contrast, GluR2-positive neurons were observed in all three layers (the *FSL*, the middle layer, *ML*, and the deep layer, *DL*) of the DCN in vehicle-treated, blast-exposed rats (*arrows* in B). The number of GluR2-positive neurons in the DCN was counted and statistically analyzed (D). Statistical significance was determined by ordinary one-way ANOVA (Tukey correction for multiple comparisons). A significantly increased number of GluR2- positive neurons was counted in the DCN of the vehicle-treated, blast-exposed group (*B+P*) compared to naive controls (*NC*, *** *p* < 0.001). Statistically fewer GluR2-positive neurons were counted in the DCN in the blast-exposed, NAC/HPN-07-treated group (*B+T*) compared to the vehicle-treated, blast-exposed group (*B*, * p < 0.05). There was no significant difference in the number of GluR2-positive neurons between the *NC* and the *B+T* groups (*p* > 0.05). Numbers in parentheses represent the number of animals evaluated in each group. The scale bar in C = 50 μm, and applies to A-C.

Previous work in primary brain tissue organotypic cultures from rats demonstrated that Arc functions to promote endocytic recycling of GluR2/3-containing receptors, leading to homeostatic decreases in AMPAR-mediated synaptic currents [57]. In light of the long-lasting reductions in Arc expression in the AC, IC, and DCN observed in vehicle-treated rats, we hypothesized that blast-induced reductions in Arc activity within these central networks might promote chronic elevations in synaptic AMPAR densities. Thus, we evaluated whether chronic reductions in Arc expression resulted in sustained elevations in GluR2 receptors in the brains of blast-exposed rats. In vehicle-treated rats, marked, blast trauma-induced elevations in GluR2 immunoreactivity were, indeed, observed in the DCN (Fig 2, Table 1). Whereas, in naive rats, GluR2 immunostaining was primarily restricted to the fusiform soma layer (FSL) of the DCN (*arrows* in Fig 2A), the blast trauma induced significant up-regulation of GluR2 expression in all three layers (FSL, middle layer [ML], and deep layer [DL]) of the DCN, resulting in prominent staining throughout (arrow in Fig 2B). Upon quantification, the number of GluR2-positive neurons in the DCN among vehicle-treated, blast-exposed rats was determined to be significantly increased compared to naive control rats (Fig 2D, *** *p* < 0.001, one-way ANOVA with Tukey post-test), such that the percent increase (86%) closely mirrored the percent decrease in Arc-positive neurons in this auditory center (75%, Fig 2D). Despite the strong correlation between reduced Arc levels and increased GluR2 levels in the DCN, no obvious increases in GluR2 immunoreactivity were detected in the AC and IC of blast-exposed rats (Table 1).

In blast-exposed rats treated with NAC/HPN-07, the number of GluR2-positive neurons in the DCN was significantly reduced compared to the vehicle-treated group (Fig 2C and 2D, * *p* < 0.05), such that there was no significant difference between naïve controls and therapeutic-treated animals at nine weeks post-blast (*p* > 0.05). Taken together, these results suggest that chronic reductions in Arc levels in central auditory structures and corresponding AMPAR accumulation in the DCN of blast-exposed rats may predispose these auditory nuclei to stimulus-independent (spontaneous) activity.

**Fig 3:**
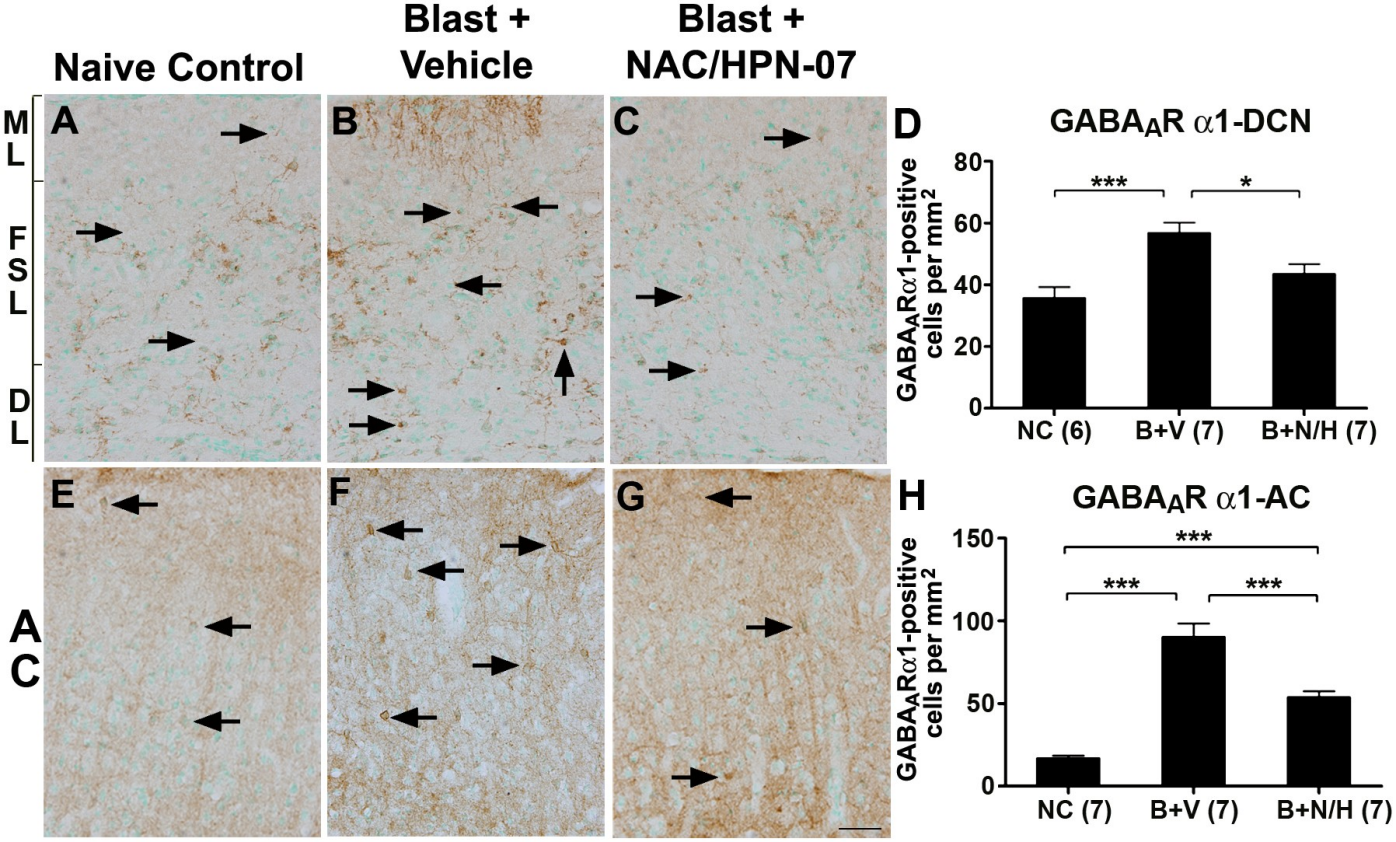
NAC/HPN-07 treatment reduced chronic blast-induced up-regulation of GABA_A_R-α1 in the central auditory system. Examples of GABA_A_R-α1 immunostaining in the DCN (A-C) and in the AC (E-G) from rats in the naive control group (*NC*, *arrows* in A and E), the vehicle-treated, blast-exposed group (*B+P*, *arrows* in B and F), and the blast-exposed, therapeutic-treated group (*B+T*, *arrows* in C and G). The number of GABA_A_R-α1-positive neurons in the DCN (D) and the AC (H) were counted and statistically analyzed. Statistical significance was determined by ordinary one-way ANOVA (Tukey correction for multiple comparisons). Significantly increased numbers of GABA_A_R-α1-positive neurons were counted in the DCN and the AC of rats in the vehicle-treated, blast-exposed group (*B+P*) compared to naive controls (*NC*, *** all *p* < 0.001). In NAC/HPN-07-treated animals (*B+T*), the number of GABA_A_R-α1-positive neurons in the DCN and AC was significantly reduced (* *p* < 0.05 and *** *p* < 0.001, respectively) compared to the vehicle-treated, blast-exposed group (*B*). There was no significant difference between the *NC* and *B+T* groups in the DCN (*p* > 0.05). Numbers in parentheses represent the number of animals evaluated in each group. The scale bar = 50 μm in G, and applies to A-C and E-G.

As maladaptive imbalances between excitation and inhibition can lead to hypersynchrony/hyperactivity [58, 59], we reciprocally examined the expression pattern of GABA_A_R-α1, a keystone subunit of fast-acting ionotropic GABA receptors, in the AC, IC, and DCN. Increases in neuronal gene expression levels for GABA_A_R subunits has previously been documented in the medial geniculate body, the cochlear nucleus, and the IC in rodent models of drug- or noise-induced tinnitus [49, 60]. In the DCN of naive controls, GABA_A_R-α1-positive staining was primarily observed in the FSL and the DL, with only a few, scattered GABA_A_R-α1-positive neurons observed in the ML (*arrows* in Fig 3A). In vehicle-treated blast-exposed animals, the number of GABA_A_R-α1-positive neurons throughout the DCN were noticeably increased (*arrows* in Fig 3B), resulting in statistically-significant elevations in immuno-positive neurons (Fig 3D, *p* < 0.001, one-way ANOVA with Tukey post-test). A similar, persistent elevation in the density of GABA_A_R-α1 immunoreactivity was observed throughout the AC at nine weeks post-blast (*arrows*, Fig 3F and 3H), which contrasted visually and quantitatively from the density observed among neurons in the AC of naïve rats, where scattered GABA_A_R-α1-positive neuronal staining was observed (*arrows*, Fig 3E).

As was observed for GluR2 immunoreactivity, therapeutic intervention with the combinatorial NAC/HPN-07 regimen significantly normalized anti-GABA_A_R-α1 staining in the DCN post-blast (Fig 3C and 3D). Statistically-significant reductions were also observed in the AC of animals treated with the active drug formulation at nine weeks post-blast (*p* ≤ 0.001, Fig 3G and 3H), albeit to a level that remained elevated relative to naïve controls.

**Fig 4:**
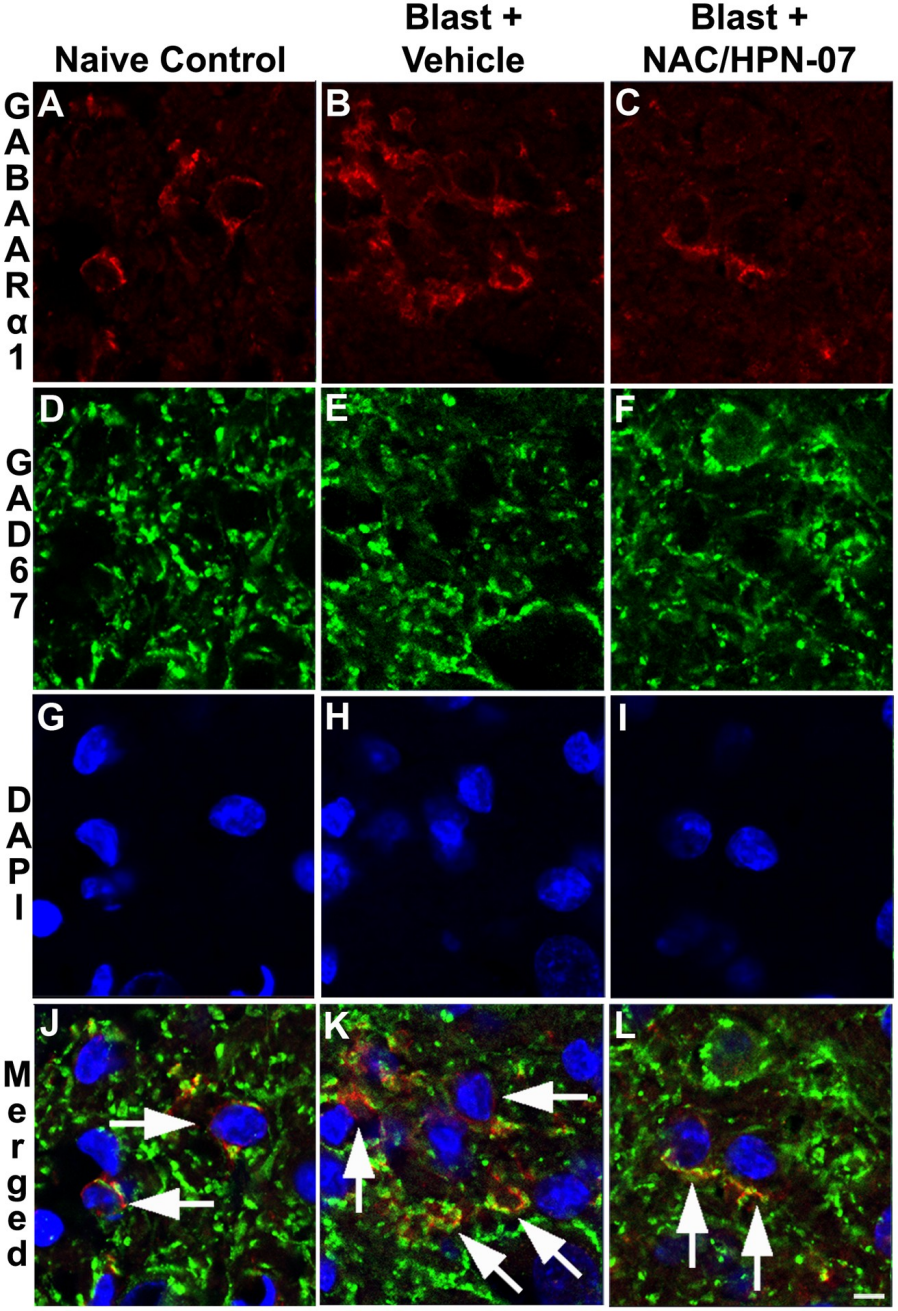
Specification of GABA_A_R-α1 positive neurons in the DCN after blast exposure. To identify the cells that persistently up-regulated GABA_A_R-α1 in the DCN at nine-weeks post-blast exposure, GABA_A_R-α1 and GAD67 (a marker for inhibitory neurons, or interneurons) co-labelling was performed in tissue sections from the naive control group (A, D, G, J), the vehicle-treated, blast-exposed group (B, E, H, K), and the blast-exposed, NAC/HPN-07-treated group (C, F, I, L). GABA_A_R-α1 and GAD67 dual-labeled neurons (*arrows* in J, K, L) are observed in the DCN of all conditions, while more dual-labeled neurons were observed in the DCN of the vehicle-treated, blast-exposed group (*arrows* in K). In these animals, all GABA_A_R-α1-positive neurons were co-labeled with GAD67, specifying them as inhibitory neurons. The scale bar = 10 μm in L, applies to A-L.

To identify the neuronal cells with persistent GABA_A_R-α1 up-regulation in the DCN, co-immunolabeling was performed with an antibody against GAD67, a marker for inhibitory neurons [61, 62]. GABA_A_R-α1and GAD67 single and dual-labeled cells were observed in the DCN of naive controls and both vehicle- and NAC/HPN-07-treated animals post-blast (*arrows* in Fig 4A–4F and 4J–4L). However, consistent with the immunostaining results in Fig 3, a greater density of GABA_A_R-α1-positive cells was observed in the DCN of blast-exposed animals in the vehicle-treated group relative to naïve controls (Fig 4A and 4B). This observation coincided with an increase in the number of GABA_A_R-α1/GAD67 dual-labeled cells in this auditory center in untreated animals (*arrows*, Fig 4K) compared to naive controls (*arrows*, Fig 4J) despite the fact that GAD67 immunolabeling patterns remained seemingly unchanged. These results suggest that the blast model induced plastic changes that promoted chronic up-regulation of GABA_A_R-α1 within the interneurons of the DCN. In animals treated with NAC/HPN-07, this aberrant elevation in GABA_A_R-α1-positive inhibitory neurons was reversed (*arrows*, in Fig 4L), resulting in an immunoreactivity pattern that mimicked that which was observed in DCN of naïve animals (Fig 4J). As increased GABA receptor expression in interneurons has been shown to reduce inhibition of principal neuronal signaling, these results would seemingly suggest that, in the absence of NAC/HPN-07 treatment, excitation signaling may be stronger or more pervasive in the central auditory pathway of blast-exposed animals [63].

**Fig 5:**
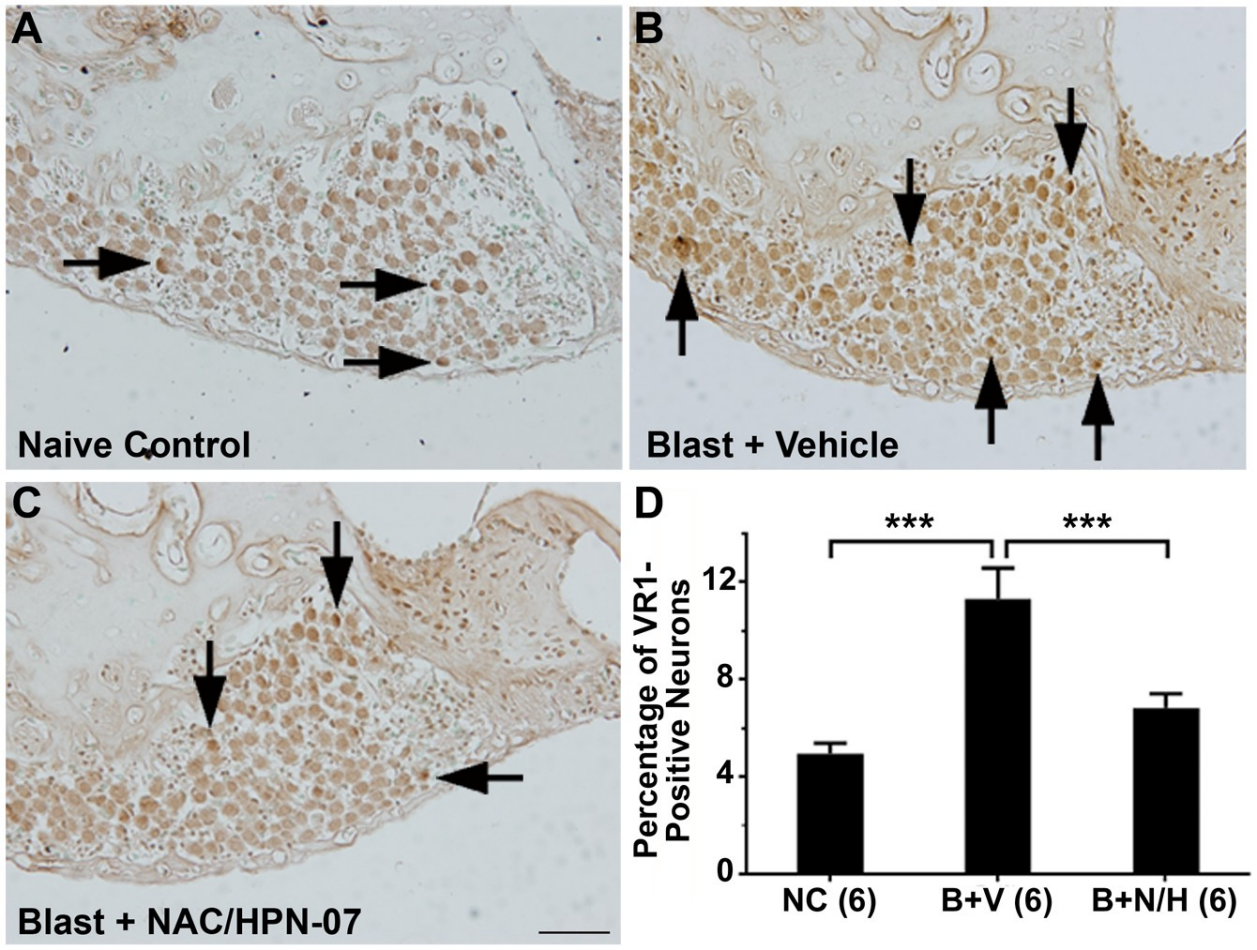
NAC/HPN-07 treatment attenuated chronic blast-induced up-regulation of VR1 in spiral ganglion neurons. Examples of VR1 immunostaining in spiral ganglia from the naive control group (A), the vehicle-treated, blast-exposed group (B) and the blast-exposed, therapeutic-treated group (C). The number of VR1-positive SGNs were counted and statistically analyzed (D). Statistical significance was determined by ordinary one-way ANOVA (Tukey correction for multiple comparisons). A significantly increased number of VR1-positive neurons were observed in the SG of the vehicle-treated, blast-exposed group (*B+P*) compared to naive controls (*NC*, *** *p* < 0.001). A significant reduction in the number of VR1-positive SGNs was observed in blast-exposed rats treated with NAC/HPN-07 (*B+T*) compared to the vehicle-treated, blast-exposed group (*B+P*, *** *p* < 0.001). There was no significant difference in the number of VR1-positive SGNs between the *NC* and the *B+T* groups (*p* > 0.05). Numbers in parentheses represent the number of animals evaluated in each group. The scale bar in C = 50 μm, applies to A-C.

In addition to central auditory pathway neuroplastic changes, chronic upregulation of vanilloid receptor 1 (VR1, also known as capsaicin receptor or TRPV1) expression in cochlear spiral ganglion neurons (SGNs) has been reported as a peripheral biomarker for tinnitus-related hyperactivity induced by an acoustic trauma [18]. Similar to published reports, we observed diffuse VR1 immunostaining among the SGNs in naïve control cochleae, with a limited number of strongly-immunoreactive SGNs observed (*arrows* in Fig 5A) [64, 65]. In vehicle-treated, blast-exposed rats, we observed sustained up-regulation of VR1 among SGNs in the first and second cochlear turns, with a higher proportion of strongly-immunoreactive SGNs evident at these anatomical positions relative to naïve controls (Fig 5A and 5B; *arrows* in Fig 5B). The number of strongly-immunoreactive SGNs across this anatomical region was counted and compared to naïve control populations (Fig 5D). From this analysis, it was determined that the blast-exposure model promoted a statistically-significant increase in the number of strongly-immunoreactive SGNs among vehicle-treated rats (*** *p* < 0.001, one-way ANOVA with Tukey post-test), with 100% of animals in this cohort exhibiting significant elevations in VR1-positive immunostaining. As disproportionately-high VR1 activation in the cochlea has been shown to result in elevated baseline firing rates among SGNs, these results suggest that the SG in vehicle-treated animals might be susceptible to chronic synaptic dysregulation post-blast [66].

We also evaluated and quantified the relative density of strongly VR1-immunopositive SGNs in blast-exposed rats that were subsequently treated with NAC/HPN-07 (Fig 5C). Therapeutic intervention with the active drug formulation significantly reduced the number of these VR1-positive neurons in the SG at nine weeks post-blast (*p* < 0.001 compared to vehicle-treated controls, one-way ANOVA with Tukey post-test, Fig 5D), such that there was no significant difference between naïve controls and blast-exposed rats treated with NAC/HPN-07 (*p* > 0.05). These results indicate treatment-specific mitigation of prolonged blast-induced VR1 upregulation among neurons in the SG after antioxidant treatment.

In summary, blast-induced changes in key neuroplasticity-related biomarkers in the peripheral and central auditory pathways provided objective evidence that the shock tube blast model induced chronic imbalances in synaptic gating factors that have previously been observed in animals with behavioral manifestations of tinnitus. Moreover, these seemingly maladaptive changes were significantly mitigated by treatment with NAC/HPN-07 post-injury, suggesting that this therapeutic intervention might short-circuit the development of this auditory disorder in blast-exposed animals.

### Blast-induced tinnitus and NAC/HPN-07 effects on tinnitus treatment are manifested in the behavioral measures

To test this hypothesis, new cohorts of animals were exposed to the same blast overpressure model, and methods and measures were employed to evaluate behavioral evidence of tinnitus in blast-exposed animals treated with either vehicle or NAC/HPN-07. To this end, a stimulus level at 80 dB SPL for the ABR amplitude measurements was selected to highlight the response from the low-SR fibers [67] that have been shown to be vulnerable to acoustic trauma relative to the high-SR fibers and cochlear sensory hair cells [22, 23]. To provide us with insights into the acoustic characteristics of the putative blast-induced tinnitus quality, we used 1/3-octave band-pass noise centered at frequencies of 9–24 kHz for tinnitus pitch-matching based on the fact that rodent models of noise-induced tinnitus have been described as “high-pitched tonal sounds” [68, 69].

Based on prior studies, tinnitus and hyperacusis are common sequelae of acoustic overexposure and are often comorbid [1, 2]. Gap-Pre-Pulse Inhibition (PPI) deficits (which are denoted by an increase in tinnitus index scores) are attributed to tinnitus [47, 49, 70], while enhanced acoustic startle amplitudes and stronger acoustic PPI values (which indicate an enhanced pre-pulse inhibition) are suggestive of hyperacusis [71–74]. The same behavioral predictors were applied in the present study to assess tinnitus- and hyperacusis-like behaviors. Behavioral testing was performed at approximately eight weeks after blast exposure on the following three groups: (1) control—20 naïve unexposed rats; (2) vehicle—20 exposed rats receiving saline; and (3) treatment—17 exposed rats receiving NAC/HPN-07. Gap detection deficits in animal models were hypothesized to indicate the presence of a tinnitus percept, consistent with the idea that a silent gap in a continuous acoustic “carrier” would be less detectable if the perceived tinnitus has similar characteristics to the carrier tone and “fills in the gap” [47]. The tinnitus index score (as a variant of gap PPI) is a robust estimator for tinnitus, meaning that this metric is not greatly influenced by some potential confounders (for details, see [49]). Here we calculated an index score from the gap PPI and the acoustic PPI (where score = gap PPI—acoustic PPI) at four targeted frequencies—i.e., 9.3, 12, 20 and 24 kHz.

**Fig 6:**
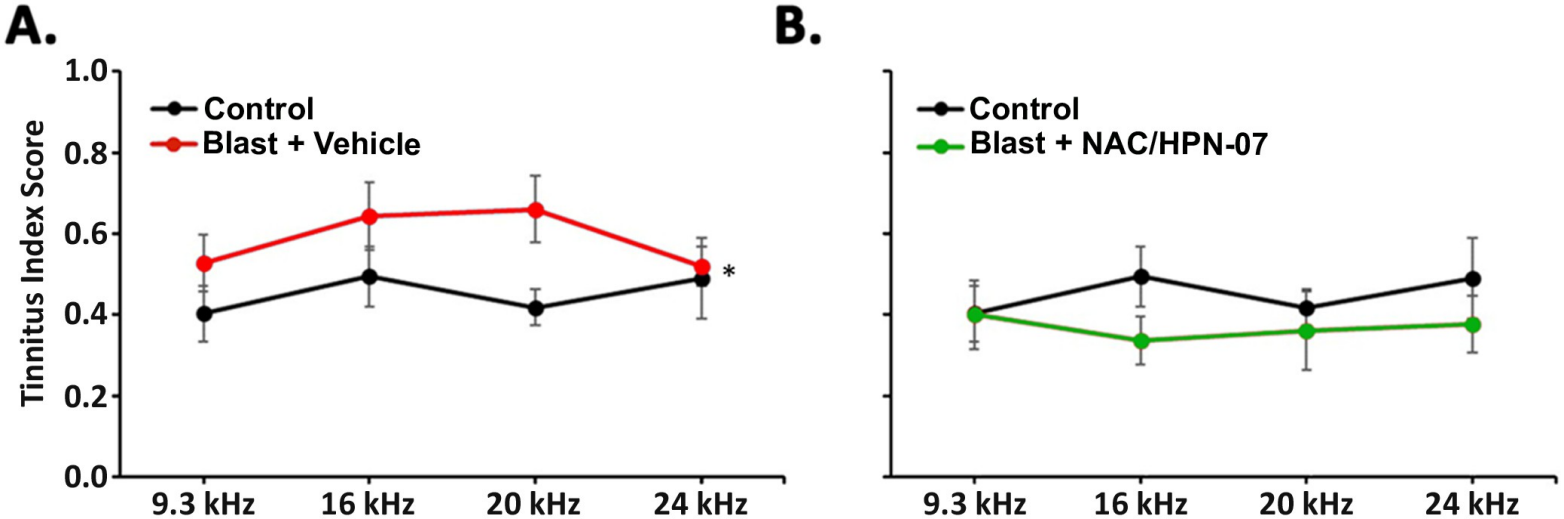
NAC/HPN-07 treatment significantly reduced the development of a chronic blast-induced tinnitus percept. Tinnitus index scores of the acoustic startle were increased in blast-exposed rats treated with vehicle (saline) but not altered in those receiving therapeutic NAC/HPN-07 treatment when compared with naïve, unexposed controls. Mean (± SE) scores are shown for each group, where *n* = 17–20 rats/group. Data were obtained at 8 weeks after blast exposure. Statistical significance by a mixed ANOVA: * *p* < 0.05, the main effect of group.

**Fig 7:**
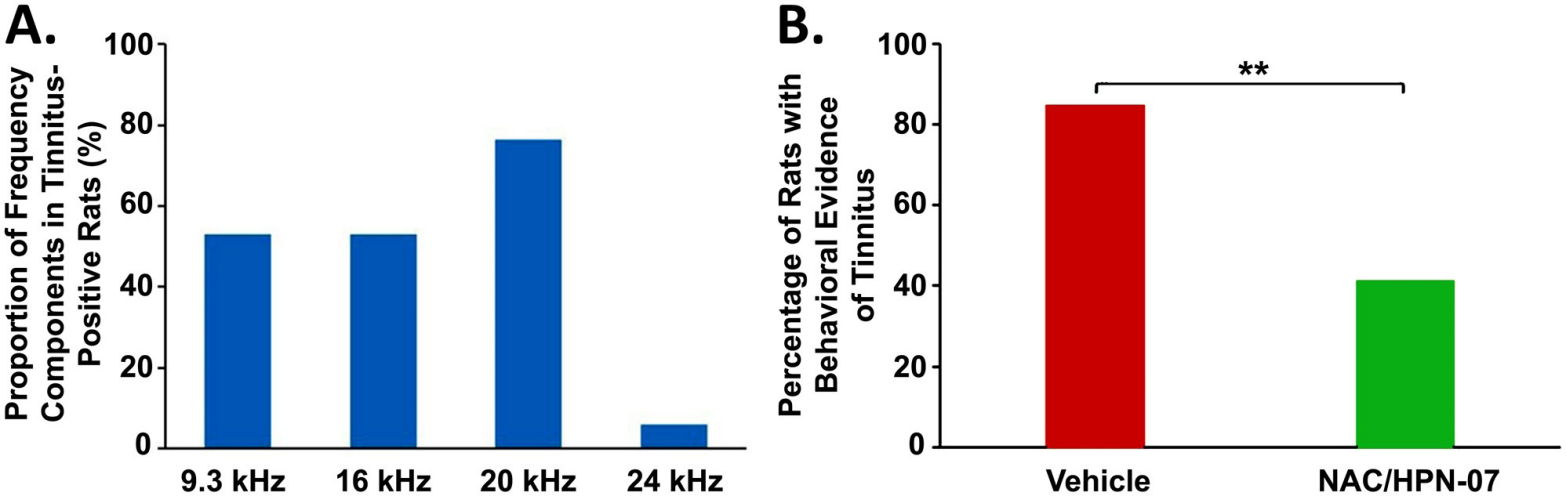
Incidence and frequency-dependent manifestations of tinnitus percept in blast-exposed rats. (A.) summarizes the incidence of tinnitus in the therapeutic-treated group in comparison to the vehicle-treated group. Data were obtained at 8 weeks after blast exposure. Statistical significance was determined by Fisher’s exact test: ** *p* < 0.01. A 50% reduction in the incidence of a chronic tinnitus percept was observed in the therapeutic treatment group relative to the vehicle treatment group. (B.) depicts the number of times that each of the four test frequencies was deemed a tinnitus percept frequency in the vehicle treatment group. These values were then expressed as a proportion of the total number of vehicle-treated rats with tinnitus. Here, “tinnitus frequency” was defined as the test frequency with an index score that exceeded the 95% confidence interval of the companion naïve control group. The blast-induced tinnitus percepts were characterized by a wide frequency range with a peak of around 20 kHz.

Overall, the index scores were significantly increased in the 9–24 kHz range in the vehicle treatment group (Fig 6A) but remained statistically unchanged in the NAC/HPN-07 treatment group (Fig 6B) when compared with those of the naive controls. Specifically, a mixed model ANOVA revealed a significant main effect of exposure group on index score (*F*_(1,114)_ = 4.508, *p* < 0.05) and a non-significant interaction of exposure group and test frequency (*F*_(3,114)_ = 1.890, *p* > 0.05) for the vehicle-treated group, suggesting that blast overpressure caused gap-detection deficits across a wide frequency range in rats. In contrast, neither a main effect of exposure group (*F*_(1,105)_ = 1.716, *p* > 0.05) or a two-way interaction of group and frequency (*F*_(3,105)_ = 0.5104, *p* > 0.05) were found to be significant for the NAC/HPN-07 treatment group, indicating that the drug formulation was effective in preventing gap-detection failures due to acoustic trauma. With a 95% confidence interval for the average control tinnitus index score determined for each test frequency (which was applied as a diagnostic criterion for tinnitus), we identified 17 out of the 20 blast-exposed animals with behavioral evidence of tinnitus in the vehicle treatment group (Fig 7A), representing an incidence of 85%, where tinnitus was characterized by the perception of multiple or overlapping tones. Fig 7B gives an overview of the frequency distribution of measured tinnitus percepts, showing a peak distribution around 20 kHz. In contrast to the high incidence proportion of blast-induced tinnitus measured among vehicle-treated, only 7 of the 17 blast-exposed subjects treated with NAC/HPN-07 (41%) developed tinnitus. This represents a 50% reduction in the incidence of tinnitus in the therapeutic treatment group compared with the vehicle treatment group (Fig 7A), where the difference in proportion was significant by Fisher’s exact test (*p* < 0.01).

**Fig 8:**
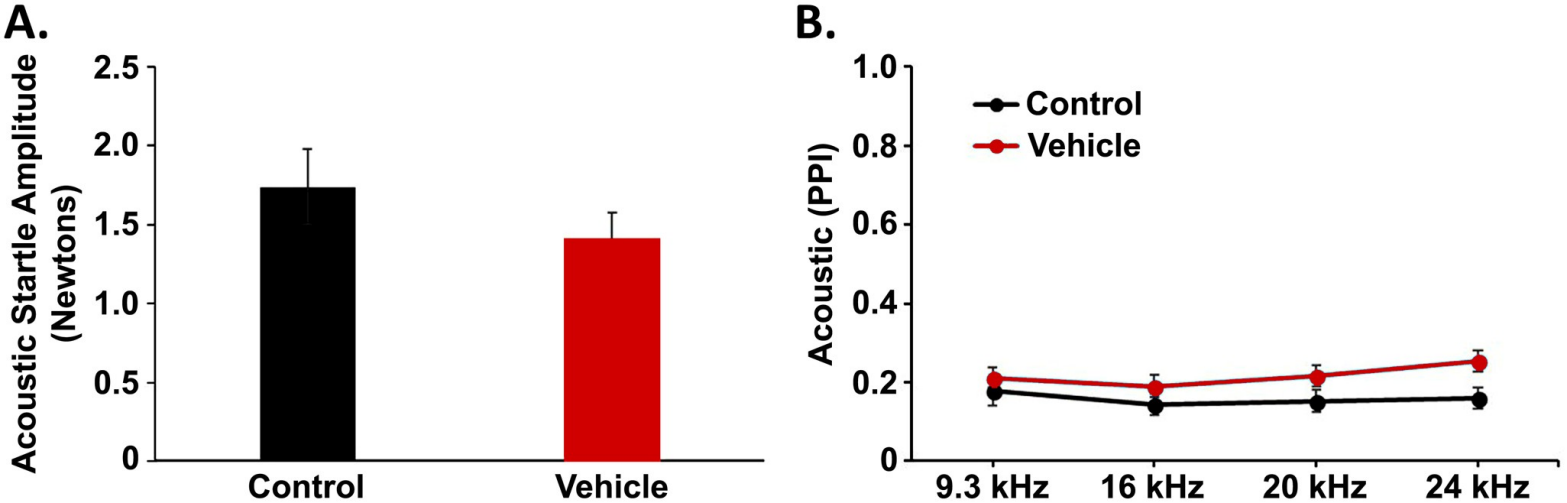
The shock tube blast exposure paradigm did not induce behavioral evidence of hyperacusis. Both acoustic startle response (ASR) and acoustic prepulse inhibition (PPI) of startle remained unchanged in blast-exposed rats that underwent vehicle treatment compared to unexposed, naive control rats, suggesting that blast overpressures did not result in hyperacusis-like behavior. Mean (± SE) values are shown for ASR (A.) and PPI (B.), where *n* = 20 rats/group. Data were obtained at 8 weeks after blast exposure.

Hyperacusis was assessed with acoustic PPI, as well as with acoustic startle response (which was conducted in a quiet background). Here, blast-exposed rats in the vehicle-treated group showed control-like responses manifested both in acoustic startle amplitude (Fig 8A) and in acoustic PPI (Fig 8B). Indeed, the comparison of the two means from the vehicle treatment and naïve control groups revealed that (1) startle amplitudes were not significantly different in a t-test (*p* > 0.05) and (2) acoustic PPI did not differ significantly in either a main effect of exposure group (*F*_(1,114)_ = 2.829, *p* > 0.05) or an interaction of exposure group and test frequency (*F*_(3,114)_ = 1.273, *p* > 0.05) in a mixed ANOVA. From these results, it was concluded that our blast exposure model did not exaggerate startle-based behavior of possible relevance to hyperacusis.

### Blast exposure resulted in ABR threshold elevations attributable to the loss of outer hair cells

ABR threshold shifts in blast-exposed animals were defined relative to pre-blast (baseline) ABR thresholds in each animal and compared to the average thresholds for age-matched naïve control animals. Blast-induced hearing loss is summarized in Table 2. Both blast-exposed cohorts showed similar degrees of frequency-dependent ABR threshold shifts when measured at eight weeks after the blast-exposure, where the threshold shifts graded in severity from low to high frequencies. The cochlear amplifier (which is an OHC-based active process) has been proposed to increase hearing sensitivity by amplifying the basilar membrane response to soft sounds [75, 76]; and as such, hearing thresholds are elevated by 40–50 dB in the absence of OHC function [77, 78]. In the present study, the mild to moderate amounts of hearing loss (up to 40 dB) observed can be assumed to arise predominately from OHC loss. Here, a mixed-design ANOVA did not reveal significant between-group (NAC/HPN-07 vs. vehicle) differences either in main effect of exposure group (*F*_(1,216)_ = 0.9747, *p* > 0.05) or in interaction of test frequency and exposure group (*F*_(3,216)_ = 0.2852, *p* > 0.05). By means of repeated observations at four and eight weeks after exposure, subsequent within-group comparisons revealed that an ABR threshold shift could be reduced by 6 dB (e.g., at 24 kHz) in the therapeutic treatment group but by only up to 3.5 dB (observed at 8 kHz) in the vehicle treatment group. Given that ABR threshold detection is associated with varying stimulus levels in 5-dB steps, the test-retest variability might be expected to be within 5 dB. As such, an attenuation of 6 dB at threshold, rather than a 3.5-dB attenuation that fell within the 5 dB ABR variability, can be reasonably regarded as a practically-important improvement in hearing level, even though both results as an estimate of within-group differences were statistically significant at *p* < 0.05 (for details, see Table 2). The findings suggest that NAC/HPN-07 may have promoted a degree of progressive threshold recovery in blast-exposed rats, as was previously observed in our previous work [41].

**Table 2:** ABR threshold shifts after blast exposure.

			Threshold shifts (dB re control mean)				ANOVA statistics	
Group	Week after blast	Number of ears	4 kHz	8 kHz	16 kHz	24 kHz	Interaction	Main effect
Placebo	4	40	9.9 ± 1.1	10.9 ± 1.3	17.5 ± 1.0	43.4 ± 2.6	*F*_(3,117)_ = 1.4076	*F*_(1,39)_ = 15.4847 ***
	8	40	8.7 ± 1.1	8.0 ± 1.4	15.8 ± 1.4	42.0 ± 2.7		
Therapeutic	4	34	6.2 ± 1.1	11.1 ± 1.4	18.4 ± 1.6	45.5 ± 2.9	*F*_(3,99)_ = 5.583 **	
	8	34	5.6 ± 0.9	7.6 ± 1.3 ^+++^	14.9 ± 1.5 ^++^	39.5 ± 3.6 ^+++^		

Values are given as mean ± SEM. Mean within-group differences in threshold shift between 4 and 8 weeks:

** *p* < 0.01,

*** *p* < 0.001 by two-way repeated measures ANOVA;

^++^ *p* < 0.01,

^+++^ *p* < 0.001 by paired t-test with use of Holm-Bonferroni correction.

### IHC ribbon loss following blast exposure results in the enhancement of the ABR wave-V/I amplitude ratio

**Fig 9:**
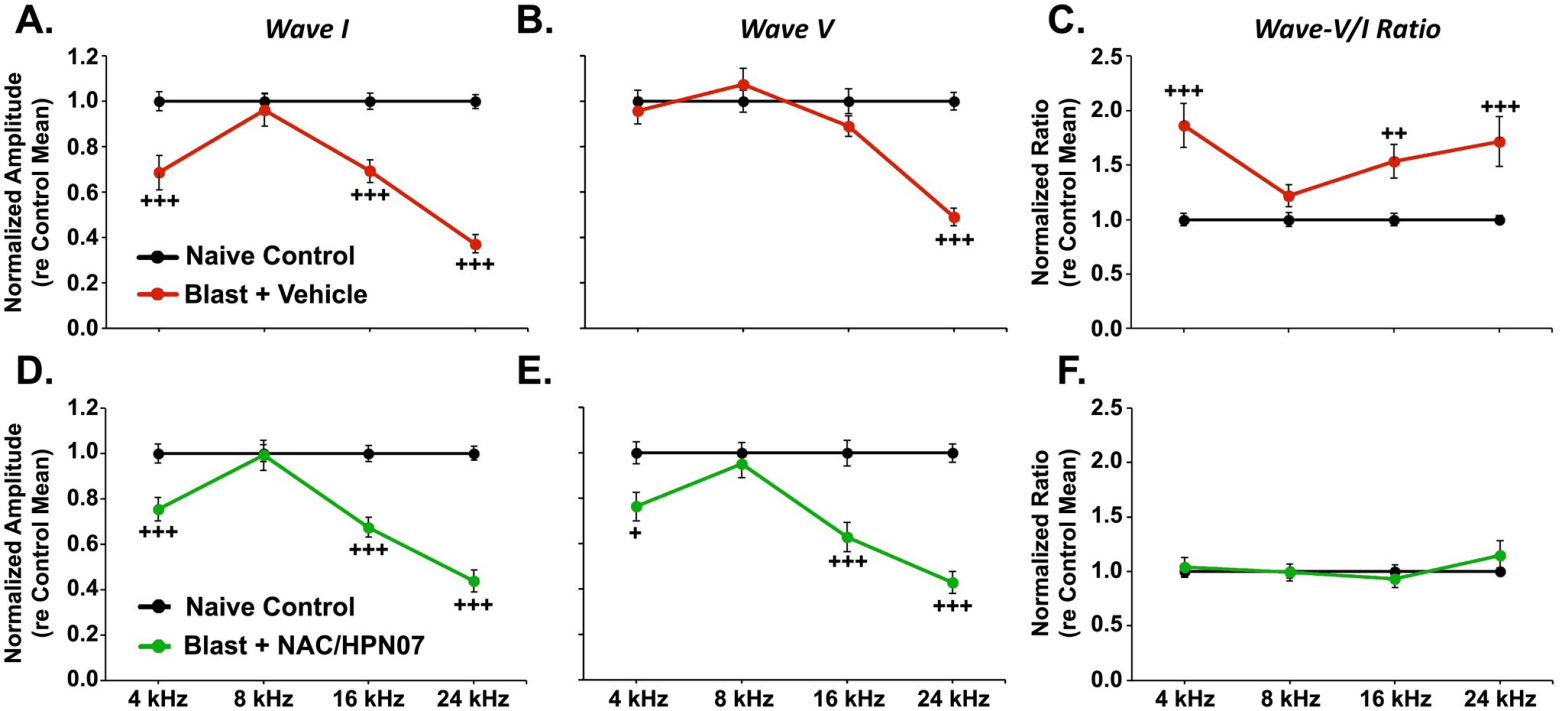
NAC/HPN-07 treatment normalized the blast-induced enhancement of the wave V-to-wave I amplitude ratio of ABRs. In vehicle-treated, blast-exposed rats, significant reductions in ABR wave-I amplitudes were measured at 4, 16, and 24 kHz (A.). Naïve control-like ABR wave-V amplitudes were measured at 4, 8, and 16 kHz (B.), thus leading to enhancement in wave-V/I amplitude ratios (C.), where 8 kHz presented an exception: no changes in ABR measurements. In the therapeutic-treated cohort, the changes in ABR wave-I amplitudes across the test frequency range (D.) were approximately equivalent to those measured for the ABR wave-V amplitudes (E.), such that the ABR wave-V/I ratio was maintained within a normal range (F.). Symbol keys in each row apply to the three panels in the same row. ABR wave-I amplitudes (A., D.), ABR wave-V amplitudes (B., E.), and wave-V/I amplitude ratios (C., F.) were normalized to naïve control means. Means (± SE) in response to tone bursts at 80 dB SPL are shown, where *n* = 34–40 ears/group. Data were obtained at 8 weeks after blast exposure. Statistical significance was determined by *t*-test with Holm-Bonferroni correction: ^+^ *p* < 0.05, ^++^ *p* < 0.01 and ^+++^ *p* < 0.001, represent the simple effect of group at different test frequencies.

The ABR wave-I and -V amplitudes and the resultant wave-V/I amplitude ratios in response to 80 dB SPL were obtained at each test frequency for each blast-exposed ear and then normalized with respect to the corresponding mean values among age-matched, naïve controls. The ABR wave I represents the summed activity of the spiral ganglion neurons (SGNs), while the ABR wave V, which is generated in the lateral lemniscus and inferior colliculus [79], reflects activity of the central auditory pathway. Here, we studied the trauma-evoked changes in auditory physiology at approximately eight weeks post-exposure, using ABR measurements. In the vehicle-treated group, we found enhancement of the wave-V/I amplitude ratios compared with naïve age-matched controls at frequencies from 4 to 24 kHz (not including 8 kHz, Fig 9C), which was attributable to the combination of severely reduced wave-I amplitudes (Fig 9A) and persistent wave-V amplitude levels despite the loss of peripheral input (Fig 9B). Based on the therapeutic effects of NAC/HPN-07 on behavioral evidence of tinnitus (see Figs 6B and 7B), we predicted normalization of the ABR wave-V/I ratio, which was, in fact, observed in the active treatment group (Fig 9F). In blast-exposed animals treated with NAC/HPN-07, the degree of the wave-I amplitude reduction (Fig 9D) was reciprocally balanced by the degree of the wave-V amplitude reduction (Fig 9E), thus maintaining normalized wave-V/I ratios. In the vehicle-treated group, a mixed ANOVA yielded a significant interaction of the exposure group and test frequency for ABR wave-I amplitude (*F*_(3,234)_ = 28.89, *p* < 0.0001) and for ABR wave-V amplitude (*F*_(3,234)_ = 22.48, *p* < 0.0001), culminating in the determination of a significant interaction for the wave-V/I ratio (*F*_(3,234)_ = 6.910, *p* < 0.001). In addition, significant simple effects were determined for wave-I amplitude at 4, 16 and 24 kHz, for wave-V amplitude at 24 kHz, and for the wave-V/I ratio at 4, 16 and 24 kHz, relative to naïve controls. In NAC/HPN-07 treated rats, the ANOVA analysis revealed a significant interaction of group and frequency both for wave-I amplitude (*F*_(3,216)_ = 29.42, *p* < 0.0001) and for wave-V amplitude (*F*_(3,216)_ = 17.18, *p* < 0.0001), each with a significant simple effect of group on the amplitudes at 4, 16 and 24 kHz relative to naïve controls. However, for the ABR wave-V/I ratios in this treatment group, there were no calculated significant differences, either in interaction of group and frequency (*F*_(3,216)_ = 1.576, *p* > 0.05) or in main effect of group on the amplitude ratio (*F*_(1,216)_ = 0.3066, *p* > 0.05).

As seen in Fig 9A–9C, we demonstrated a link between hearing loss (seen as a reduction in wave-I amplitude) and an imbalanced enhancement in the ABR wave-V/I amplitude ratio, in agreement with the view that, after loss of peripheral input, there are compensatory plasticity changes in the central auditory pathway that maintain wave-V amplitudes [10, 29, 80]. Furthermore, our present results provide physiological evidence that NAC/HPN-07 could counteract this apparent central neural dysfunction in response to reduced cochlear neural output (Fig 9D–9F).

Recent work shows that cochlear synaptopathy (known as the loss of synapses between SGNs and IHCs) may be crucial for the compensatory hyperactivity that drives gain in central auditory pathways [10, 29]. It has been reported that a reduction in wave-I amplitude of the suprathreshold ABRs can reveal cochlear synaptopathy [20]. When running a mixed ANOVA, where “group” was a between-subjects factor and “frequency” was a within-subjects factor, we did not find a smaller reduction in mean amplitude of the wave I at each test frequency in the treatment group as compared with the nontreatment group (Fig 9A and 9D). Given this issue, “time” was also treated as a within-subjects factor (as with “frequency”) to carry out a two-way repeated measures ANOVA when testing for changes in wave-I amplitude from four to eight weeks after exposure in each exposed group (in which the same subjects underwent repeated measurements over time). The repeated measures design can remove the between-subjects variability, thus leading to an increase in the power of the test to detect significant differences between means from the same individuals, e.g. at either different time points or under different conditions.

**Fig 10:**
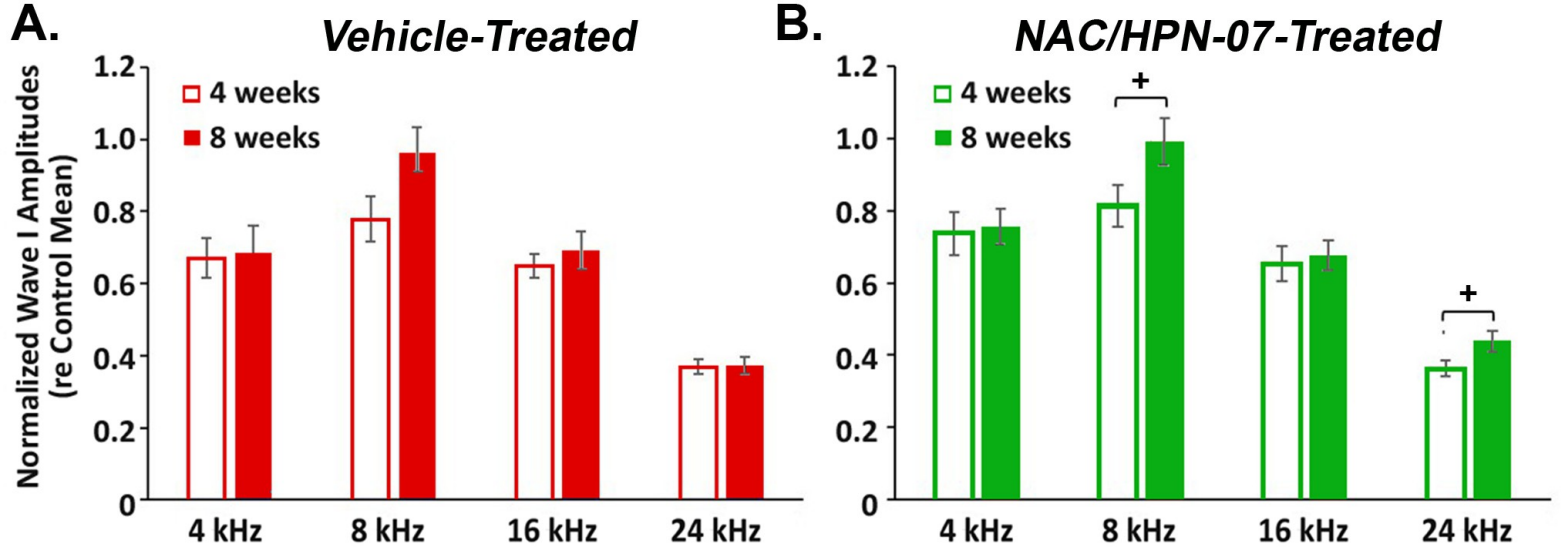
Time-dependent effects of treatment on ABR threshold sensitivity. After initial threshold stabilization, the reduced amplitudes of ABR wave I progressively recovered with increasing post-blast exposure time in the NAC/HPN-07 therapeutic treatment group (B.) but not in the vehicle treatment group (A.). ABR wave-I amplitudes were normalized with respect to mean values in age-matched, unexposed naïve controls. Mean (± SE) amplitudes in response to tone bursts at 80 dB SPL are shown, where *n* = 34–40 ears/group. Data were obtained at 4 and 8 weeks after blast exposure, respectively. Statistical significance was determined by paired *t*-test with Holm-Bonferroni correction: ^+^ *p* < 0.05, the simple effect of time at 8 and 24 kHz.

As shown in Fig 10, animals treated with the active therapeutic showed a significant improvement in wave-I amplitudes at 8 and 24 kHz over the interval between four and eight weeks after blast, suggesting a beneficial effect of NAC/HPN-07 on cochlear synaptopathy (Fig 10B). This was confirmed by a test of significant simple effects following a significant two-way interaction by ANOVA (*F*_(3,99)_ = 4.2771 *p* < 0.01). In contrast, no significant change in wave-I amplitudes over this same time period post-blast was found at any test frequency in vehicle-treated animals (Fig 10A), though time and frequency yielded a significant interaction in an overall ANOVA (*F*_(3,117)_ = 3.455, *p* < 0.05).

**Fig 11:**
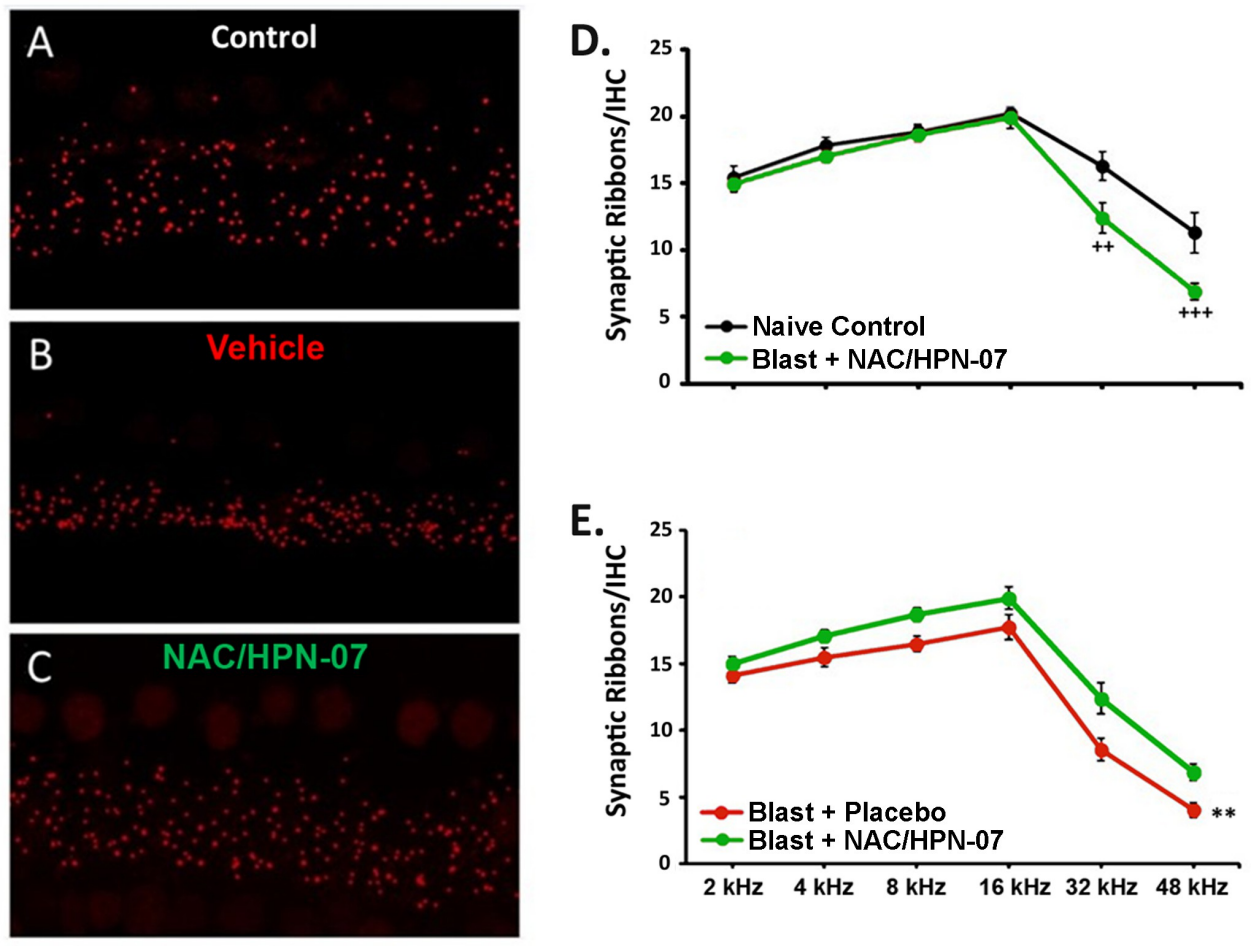
NAC/HPN-07 treatment rescued the blast-induced loss of IHC afferent synapses. A.–C. represent maximum projections from confocal z stacks of the IHC area in the 16 kHz region immunolabeled with antibody against CtBP2 (*red*) to show synaptic ribbons. D.-E. summarize mean (± SE) counts of presynaptic ribbons per IHC at each of the tonotopic positions for animals in each experimental cohort, as indicated in the symbol keys, where *n* = 12–23 ears/group. Data were obtained at approximately nine weeks after blast exposure. Statistical significance was determined by *t*-test with Holm-Bonferroni correction: ^*++*^ *p <* 0.01 and ^*+++*^ *p <* 0.001, representing the simple effect of group at 32 and 48 kHz, respectively; by a mixed ANOVA: ** *p* < 0.01, the main effect of group.

To more directly assess the potential for blast-induced cochlear synaptopathy and the positive therapeutic effect of NAC/HPN-07 on this pathophysiological response, we selected 12 ears from the naïve control group, 21 ears from the vehicle treatment group, and 23 ears from the NAC/HPN-07 treatment group for histological evaluation of IHC ribbon synapse densities. In maximum projections from confocal z-stacks, synaptic ribbons in IHCs appeared as pre-synaptic C-terminal binding protein 2 (CtBP2)-positive (*red*) puncta, as illustrated in Fig 11A–11C. The loss of ribbons was clearly visible in immunolabeled cochlear tissues from vehicle-treated, blast-exposed animals (Fig 11B). Grossly, this synaptopathy was seemingly attenuated or rescued in blast-exposed NAC/HPN-07-treated animals (Fig 11C).

To confirm these observations, mean ribbon counts per IHC were computed, the results from which are shown in Fig 11D and 11E. In NAC/HPN-07-treated rats, the synapse loss was statistically distinguishable only at 32 and 48 kHz (the extreme cochlear base), whereas the recovery was significant over the whole range of test frequencies from 2 to 48 kHz relative to vehicle-treated animals. For the comparison between NAC/HPN-07-treated ears and naïve control ears, following a significant two-way interaction by ANOVA (*F*_(5,155)_ = 4.590, *p* < 0.001), the simple effect of group proved to be significant at 32 and 48 kHz. For the comparison between NAC/HPN-07-treated ears and vehicle-treated ears, following a non-significant interaction (*F*_(5,210)_ = 1.628, *p* > 0.05), the main effect of group collapsing across frequency turned out to be significant in a mixed ANOVA (*F*_(1,210)_ = 10.20 *p* < 0.01). Taken together, the histological results demonstrated that NAC/HPN-07 had a positive treatment effect on cochlear synaptopathy. As shown in Table 2, the difference in ABR threshold shifts between these two cohorts of blast-exposed animals, however, was not statistically significant.

**Fig 12:**
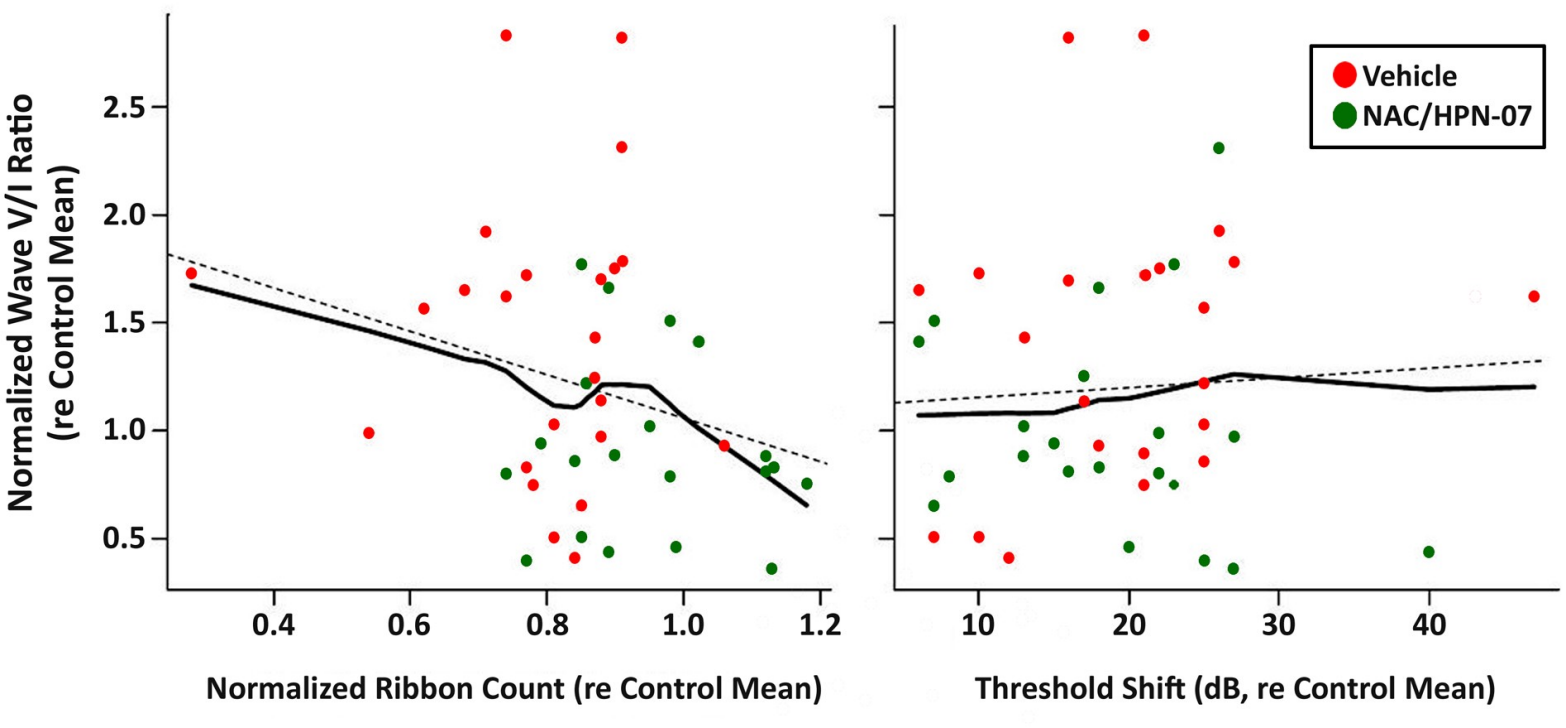
Tinnitus-related enhancement of ABR wave V/I ratios correlated with IHC de-afferentation. Loess regression suggested that the enhancement of the wave V-to-wave I ratio after blast injury is likely due to loss of cochlear synapses rather than damage to cochlear sensory cells (see Results). Loess curves were fitted to the empirical data in a scatterplot of wave-V/I ratio vs. either ribbon count (A.) or threshold shift (B.) with a smoothing span = 0.75 and a polynomial degree = 2 (quadratic). ABR wave-V/I ratios and ABR threshold shifts, as well as IHC presynaptic ribbon counts, were averaged in blast-exposed ears from each vehicle (*red dots*)- or therapeutic (*green dots*)-treated animal (*n* = 44) for frequencies from 4–32 kHz and then normalized to mean values from naïve, unexposed control ears (*n* = 12). Data were obtained at 8–9 weeks after blast exposure. Loess curves and linear trendlines (that the loess fit captures in the data) were depicted by solid lines and dashed lines, respectively.

To determine whether an enhancement of the ABR wave-V/I ratio is ascribable to a reduction in synaptic ribbon counts and/or an elevation in cochlear ABR thresholds, we applied the loess method to model such a relationship; that is, determination of whether ribbon counts or threshold shifts might serve as a predictor variable in order to observe the effect on an outcome of the wave-V/I amplitude ratio. Here, the ABR wave V/I ratio and the number of IHC ribbons were averaged for frequencies from 4–32 kHz in each blast-exposed ear and were normalized to their respective naïve control means. This process aimed to ensure that the physiological and histological data could correspond to each other in the regression model, especially in terms of the frequency-specific effect on variables. The loess regression fitted a smooth curve through points in the scatter plot of the wave-V/I ratio vs. the ribbon count (Fig 12A) or the threshold shift (Fig 12B) to reveal a potential relationship between variables as well as capturing a trend in data that might be depicted by the slope of a trendline. Where, the slope of the V/I ratio with ribbon count was far from zero, the slope relating the V/I ratio to ABR threshold shift was near zero. Thus, the loess curves revealed a dependence of elevated ABR wave-V/I ratios on loss of IHC ribbon synapses, with no clear dependence on ABR threshold shift (due to OHC loss). Therefore, these results suggest that cochlear synaptopathy, rather than direct OHC damage, was key to the development of disproportionate activity changes in the blast-exposed rats.

### ABR wave-V/I amplitude ratio as an objective marker for the identification of tinnitus

According to one widely-accepted view regarding the central tinnitus generator, tinnitus is considered to arise from a compensatory increase in gain (or neural amplification) in the central auditory system in response to the loss of peripheral input from the cochlea [11, 81]. In this model, the suprathreshold amplitudes of wave I of ABRs are predicted to be significantly reduced, revealing cochlear synaptopathy [20, 29], while the amplitudes of wave V of ABRs are increased or remain unaltered, suggesting a net gain in neuronal signaling in the auditory brainstem [10, 29, 80]. As a result, the amplitude ratio of ABR wave V to wave I might be predicted to represent a functional correlate of tinnitus.

**Fig 13:**
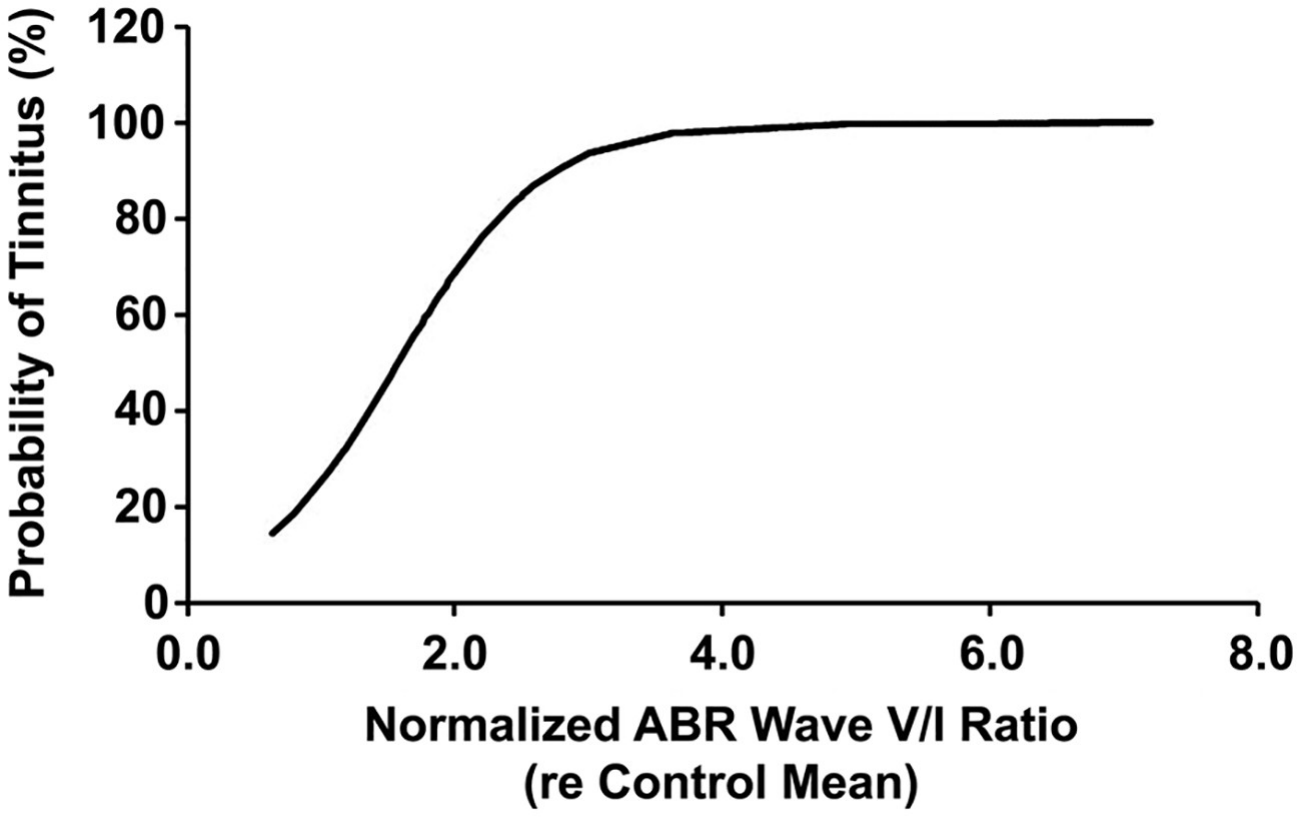
Predicted probabilities from logistic regression modeling of tinnitus relative to ABR wave V/I amplitude ratio. Observed ABR data were extracted from Fig 9C and 9F, and corresponding behavioral binary data coded as 1 (presence of tinnitus) or 0 (absence of tinnitus) were extracted from Fig 7B. Abscissa depicts the maximum ratio V/I across frequencies and ears for each subject.

In the present study, we used a binary logistic regression to find the best fitting model to determine the relationship between the presence/absence of tinnitus and the ABR wave V/I ratio, resulting in equation that can be used to predict the probability of a chronic tinnitus percept from the wave-V/I ratio. Here, “tinnitus” is a dichotomous variable with only two possible outcomes—yes and no (coded as 1 and 0)—measured in the PPI behavioral model, while “wave-V/I ratio” is a measurement variable whose value denotes the maximum value among 8 measurements (i.e., 2 ears × 4 test frequencies × 1 stimulus level) in each individual subject. Fig 13 shows the logistic regression curve that predicts the probability (ρ) of tinnitus occurrence with a given ratio $\frac{V}{I}$, where the fitted equation is: $\rho =\frac{e^{-2.9599+1.8767\;(\frac{V}{I})}}{\text{1}+e^{-2.9599+1.8767\;(\frac{V}{I})}}$. As statistical tools to estimate the difference between nested models, the likelihood ratio test and Wald test yielded *p*-values of 0.000759 (χ^2^(1) = 11.34) and 0.0151 (χ^2^(1) = 5.8947), respectively, suggesting that the ABR wave V/I amplitude ratio contributes significantly to the prediction of tinnitus. Also, as a measure of goodness of fit, the Hosmer–Lemeshow test showed a non-significant result (χ^2^(34) = 38.0693, p = 0.2893), providing evidence that the model was a good fit. The Hosmer–Lemeshow test is a formal test of the null hypothesis that the fitted model is correct, and its output is a *p*-value—a number between 0 and 1, with higher values (typically > 0.05) indicative of a better fit. Further checks of model fit were carried out on the model’s predictive power by inspection of statistics of classification table, ROC curve, pseudo-*R*^2^.

Here, the model correctly predicted 76% of tinnitus cases when evaluated by the classification table using 0.50 as the cutoff, while the model’s ability to discriminate between positive and negative cases was 0.81 when quantified by the area under the ROC curve. In terms of *R*^2^ measures, the model had a McFadden’s *R*^2^ of 0.24, a Cox & Snell’s *R*^2^ of 0.26 and a Nagelkerke’s *R*^2^ of 0.36, respectively. Basically, values of 0.2 to 0.4 for the McFadden’s *R*^2^ represent excellent model fit. In essence, all the statistics (as summarized in Table 3) for measures of fit for logistic regression verified that the model fit a given set of observations extremely well. Thus, our regression analysis indicates that the ABR wave V/I amplitude ratio might be used to diagnose tinnitus in animals exposed to an acoustic blast trauma.

**Table 3:** Measures of fit for logistic regression.

Measures	Statistics					
	Chi-square	Degree of freedom	P-value	Accuracy	Area under curve	Pseudo R-square
***Likelihood ratio test***	**11.3392**	**1**	**0.0007**			
**Wald test**	**5.8947**	**1**	**0.0151**			
**Hosmer-Lemeshow test**	**38.0693**	**34** *	**0.2893**			
**Classification table**				**0.7567** ^+^		
**ROC curve**					**0.8076**	
**McFadden’s *R***^**2**^						**0.2363**
**Cox and Snell’s *R***^**2**^						**0.2639**
**Nagelkerke’s *R***^**2**^						**0.3633**

* The groupings but not subgroups of size ten.

^+^ The cutoff value is 0.50.

## Discussion

Although the exact mechanism underlying tinnitus is unknown, the “central gain theory” has become a widely accepted view, which proposes that tinnitus results from a maladjusted increase in the “gain” of the central auditory system to compensate for loss of cochlear sensory input (for review, see [11]). Recent work has suggested that primary cochlear neuropathy, rather than hair cell loss, is a critical factor for the generation of compensatory central response patterns in the auditory pathway after acoustic overexposure [29]. In the context of peripheral auditory neuropathy (as evidenced by degeneration of cochlear nerve terminals in the IHC area and a commensurate reduction in the amplitude of ABR wave I), the maintenance of ABR wave-V amplitudes has been observed and interpreted as a sign of a maladaptive central gain increase [10, 80]. The wave-V/I amplitude ratio of ABRs, therefore, has gained attention as a potential measure of central gain enhancement that has been linked to tinnitus [10, 30].

Our comparative analyses from the control and vehicle groups share similarities with these previous findings; namely, blast-induced cochlear neuropathy (seen as a reduction in the number of IHC ribbons in Fig 11B and in the suprathreshold amplitude of ABR wave I in Fig 9A) leads to an elevation in the ABR wave amplitude ratio (Fig 9C), corresponding with an increase in behavioral evidence of tinnitus (as documented by an increase in PPI ratio in Fig 6A). Further, our correlation results showed that blast-induced elevations in the ABR wave-V/I amplitude ratio were closely associated with IHC ribbon loss (Fig 12A) but independent of ABR threshold shifts arising from OHC loss (Fig 12B).

Currently, there is no *objective test* to confirm the existence of tinnitus. Here, we constructed a binary logistic regression equation to model the probability for the occurrence of tinnitus as a function of the ABR wave-V/I amplitude ratio (Fig 13). To assess the adequacy of the fitted model, we implemented two categories of model fit tests: (1) measures of predictive power (e.g., classification table, ROC curve, pseudo-*R*^2^) and (2) measures of goodness of fit (e.g., Hosmer-Lemeshow test). The fit statistics (Table 3) suggest that the logit function is a correctly specified link function, and the model structure gives a good fit for the observed data. Given the fact that (1) tinnitus observed here was manifest as a noise-like (or multi-tonal) sound (i.e., lacking frequency-specific properties, Fig 7B), and (2) the PPI paradigm adopted here as a behavioral test for tinnitus cannot be used to determine the loudness of tinnitus (for review, see [82]), the current model exclusively focused on the “presence or absence” of tinnitus and is incapable of estimating the pitch and volume of tinnitus, which remain compelling issues for future computational models of tinnitus.

Using this model of blast-induced tinnitus, we found that therapeutic intervention with NAC/HPN-07 significantly reduced the incidence of tinnitus (Fig 7A) in the context of completely normalized ABR wave-V/I ratios (Fig 9F) and partial preservation of IHC synapses (Fig 11D) with marked improvements in ABR thresholds (Table 1) and wave-I amplitudes (Fig 10B). Prior studies have shown that acoustic traumas cause immediate, permanent loss of IHC ribbon synapses despite complete threshold recovery and lack of hair cell damage [20, 21, 23], where synapse counts at time zero post-trauma are similar to those seen at eight weeks post-exposure [20]. Despite the immediacy and permanence of IHC de-afferentation documented in prior studies, we observed treatment-specific reductions in ribbon synapse loss when treatment was initiated one-hour post-blast. NAC and HPN-07 have been shown to have protective effects on cochlear neurons following both noise- and blast-induced acoustic traumas [40–43]. Thus, NAC and HPN-07 may exert their therapeutic effect on peripheral afferentation by targeting key neuronal survival pathways (such as the Ras-ERK signaling cascade) that play an important role in axon survival and neurite outgrowth. Alternatively, the progressive ABR threshold recovery that we observed in NAC/HPN-07-treated animals (Table 2) may reflect an inducible cochlear repair mechanism that mitigated the behavioral and neurological changes induced by blast in a manner either complementary to, or independent of, the therapeutic effects on IHC synaptopathy.

NAC’s efficacy as a neuroprotectant is well-established [33, 83, 84]. HPN-07, a "second generation" spin-trapping agent, is a disulfonyl derivative of PBN, which has been shown to induce neurite outgrowth cells through activation of the Ras-ERK pathway and protein kinase C [85, 86]. Thus, HPN-07 may also possess pro-neuritogenic properties. This putative attribute may explain the unexpected long-term (six-month) and progressive recovery in auditory function that was observed in our previous studies among noise-exposed chinchilla treated with HPN-07 monotherapy [42] and may constitute a candidate mechanism underlying the prevention or pharmacological reversal of tinnitus observed in the present study. It is noteworthy that 28.6% of the rats in the active treatment group presented with average ribbon synapse densities that were even greater than those observed in naïve control cochleae across the same anatomical span (Fig 12A). This result bears further investigation and illustrates an inherent limitation of the current study: the lack of a non-blast-exposed control group that was administered NAC/HPN-07 and evaluated in parallel with naïve controls and blast-exposed cohorts for both behavioral and histological metrics, a knowledge gap that will be the focus of future studies.

To explore molecular mechanisms of tinnitus, we surveyed the expression levels of neuroplasticity-related biomarkers: (1) VR1 in the SG and (2) Arc, GABA_A_R-α1 and GluR2 in the AC, IC and DCN. We observed that, in untreated animals, blast overpressures caused chronic downregulation of Arc in the central auditory pathway, which is consistent with prior studies on tinnitus [26, 54, 56], [87]. We also observed chronic upregulation of GABA_A_R-α1 and GluR2 in these brain nuclei and elevated levels of VR1 in the SG.

Prior work suggests that cochlear neuropathologic changes, such as loss of IHC synapses and changes in BDNF or c-Fos levels in the SG, trigger downregulation of Arc in central auditory pathways, which has been correlated with tinnitus [26, 54, 55]. It is noteworthy that elevated VR1 activity has been shown to both reduce OHC activity and enhance baseline spikes of SGNs [65], [66], which may contribute to tinnitus-related background activity [88].

Under normal central conditions, salient stimuli up-regulate Arc activity in neurons to promote post-synaptic AMPA receptor endocytosis to govern and maintain synaptic strength [89, 90]. Therefore, blast-induced downregulation of Arc would be predicted to result in a net increase in GluR2 levels in excitatory principal neurons, as was observed here in the DCN (Figs 1 and 2). This reduction in Arc expression and elevation in AMPAR levels may, in turn, promote neuronal hyperexcitability that is implicated in tinnitus-like behavior [91–93]. Indeed, we observed evidence for blast-induced elevations in GAP43 levels in the DCN (and NAC/HPN-07-specific mitigation) at nine weeks post-exposure (S1 Fig), which has intriguing implications for tinnitus-related maladaptive plasticity in the DCN [94–98].

Reciprocal to Arc reductions, we also observed blast-induced elevations in GABA_A_Rα-1 levels in the DCN and AC. We found that, while GABA_A_R-α1 immunolabeling was more prevalent in the DCN, this immunoreactivity pattern was restricted to GAD67-positive neurons (Fig 4), suggesting increased silencing of inhibitory neurons, which could result in disproportionate or dysregulated excitation [63]. An alternative explanation is that an increase of GABAergic neurons in the DCN is representative of trauma-induced neurogenesis. Prior studies have demonstrated that cochlear de-afferentation can induce reactive proliferation in the CN [99–101]. Although the significance of neurogenic outcomes is challenging to predict, some new GABAergic neurons may become integrated within the CN signaling network and promote either adaptive or maladaptive plasticity. At face value, neurogenic integration of CN interneurons would be predicted to increase central inhibition, thus opposing central hyperactivity. However, recent work in feline models demonstrates that these central neurogenic events are accompanied by pervasive loss of the membrane-bound KCC2 potassium-chloride cotransporters [101]. The resultant loss of these transporters among GABAergic neurons in the CN would be predicted to cause their depolarization and potentially induce excitatory effects of GABA on membrane potentials.

Considering the close relationship between the imbalanced expression patterns of gating factors in the auditory system and tinnitus, recent work suggests that animals exposed to shock-blast waves are particularly prone to develop tinnitus [87], a conclusion that is supported by *approximately* 85% probability of tinnitus observed in this study. In light of the prevalence and multi-site manifestations of blast-induced histopathological abnormalities, it is remarkable that NAC/HPN-07-treatment significantly normalized the aberrant biomarker expression patterns in the auditory system, suggesting that this treatment strategy can reduce, or perhaps reverse, both primary neurodegeneration and excitotoxic trauma in cochleae and prevent or attenuate maladaptive neural plasticity in central auditory pathways.

## Supporting information

S1 File Supplemental results and methods. (DOCX)

S1 Fig NAC/HPN-07 treatment reduced the blast-induced up-regulation of GAP-43 in the central auditory system. (TIF)

S1 Raw Images (PDF)
