# Supplementary material for: Electrophysiological assessment and pharmacological treatment of blast-induced tinnitus
Source: PLoS One. 2021 Jan 7;16(1):e0243903. doi: 10.1371/journal.pone.0243903 (PMC7790300; doi:10.1371/journal.pone.0243903)
Supplement: S1 File — (DOCX) [file pone.0243903.s001.docx]

**S1 File. Supplemental Results and Methods**

***Supplemental Results***

**Antioxidants reduce chronic blast-induced up-regulation of GAP-43 in the central auditory system**

GAP-43 is a marker of neuroplasticity and has been shown to be upregulated in auditory nuclei in response to acoustic trauma (Illing and Horvath, 1995; Bilak *et al.*, 1997; Michler and Illing; 2002; Illing *et al.*, 2005; Meiding *et al.*, 2006; Kraus *et al*., 2011). GAP-43 immunostaining was examined in the DCN, IC, and AC among each of the experimental cohorts at nine weeks post-blast. In comparison to naïve controls (*NC*, Fig S1, A and F), the blast-exposure resulted in noticeably greater GAP-43 immunoreactivity in the DCN and IC regions of the central auditory system in vehicle-treated animals (*arrows* in Fig S1, B and G). In the AC, GAP-43 immunostaining was only slightly elevated in blast-exposed rats (Fig S1, K and L). The increased immunoreactivity observed in the IC and DCN among animals in the vehicle-treated blast-exposed cohort, however, was diffusely distributed, confounding formal quantification of neurons expressing elevated levels of GAP-43.

**S1 Fig.** **NAC/HPN-07 treatment reduced the blast-induced up-regulation of GAP-43 in the central auditory system.** Examples of GAP-43 immunostaining in the DCN (A-C), the IC (F-H) and the AC (K-M) from the naive control group (*NC*; A, F, K), the vehicle-treated, blast-exposed group (*B+P*; B, G, L) and the blast exposed, therapeutic-treated group (*B+T*, C, H, M). More GAP-43 immunostaining was observed in the DCN and the IC of the vehicle-treated, blast-exposed animals (*arrows* in B and G) compared to the naive control group (A and F). Less GAP-43 immunoreactivity was observed in the DCN and IC of the NAC/HPN-07-treated animals (C and H) compared to the corresponding vehicle-treated animals (B and G) at nine weeks post-blast. No significant elevations in GAP-43 levels were observed in the AC in either blast-exposed cohort (K-P, all *p* > 0.05). Flash-frozen brain tissues were microdissected for preparation of tissue homogenates from the DCN, IC, and AC, and equivalent amounts of total protein in the resultant extracts were subjected to immunoblot evaluations with antibodies directed against GAP-43 and the house-keeping gene, GAPDH, examples of which are shown in D, I, and N. Relative levels of GAP-43 expression in each sample were determined by densitometric analyses, using NIH Image J software and GAPDH as an internal loading control. Densitometric analyses from technical and biological replicates for each treatment group were averaged and graphed (E, J, and O). In the DCN and IC, blast-exposure promoted chronic, statistically-significant increases in total GAP-43 levels in vehicle-treated rats (* and **, *p* < 0.05 and 0.01, respectively, E and J). No such relative elevations were observed in the AC (*p* > 0.05, O). In blast-exposed rates treated with NAC/HPN-07, statistically significant reductions in total GAP-43 levels were observed in both the DCN and IC (# and ##, *p* < 0.05 and 0.01, respectively, in comparison to vehicle-treated controls, E and J). The scale bar = 20 μm in I, and applies to A-C, F-H, and K-M.

To confirm the general increases in GAP-43 immunoreactivity observed by immunohistochemistry, immunoblot evaluations of GAP-43 levels were conducted among tissue samples from the DCN, IC, and AC. Densitometric evaluations of GAP-43 levels from the DCN and IC of vehicle-treated animals exposed to blast confirmed significant increases in the total relative levels of this neuroplasticity factor (~1.6 ± 0.38 and ~1.7 ± 0.32, respectively) compared to normal control levels in these auditory centers of the brain (Fig S1, D-E and I-J).

Conversely, GAP-43 immunostaining in the IC and DCN were seemingly normalized in blast-exposed animals treated with NAC/HPN-07 (Fig S1, C and H). Immunoblot evaluations of relative GAP-43 levels in these animals revealed that GAP-43 levels were, indeed, grossly normalized in the DCN and IC, such that no significant increases in GAP-43 expression were detected in either of these brain regions at this time point post-blast (1.0 ± 0.39 and 0.97 ± 0.2, respectively, Fig S1, D-E and I-J). Consistent with the stereotactic histologic observations, no significant differences in total GAP-43 levels were observed in the AC for either vehicle-treated or NAC/HPN-07-treated animals at nine-weeks post-blast (Fig S1, N and O, all *p* ≥ 0.05).

***Supplemental Methods***

***Immunohistology:***

Blocked and permeabilized brain tissue sections were prepared from naïve controls and blast-exposed rats treated with either vehicle or NAC/HPN-07 and subjected to immunohistochemical staining for GAP43 as described in the main body of the text. Sections were incubated with mouse anti-GAP-43 (1:5000, clone 9-1E12, EMD Millipore, Billerica, MA, catalog# MAB347) overnight at room temperature. After washing with PBS/T, biotinylated horse anti-mouse IgG (1:200, Vector Laboratories, Inc. Burlingame, CA) was applied to the slides for one hour at room temperature, and Vectastain ABC and DAB kits (Vector Laboratories, Inc. Burlingame, CA) were used for the immunolabeling visualization. Immuno-positive cells exhibited a brown reaction product at the sites of the target epitopes.

***Immunoblot evaluations:***

Brains harvested at nine-weeks post-blast were cryosectioned in a coronal plane at a thickness of 50 µm. Tissue sections from the DCN, IC, and AC were microdissected on ice under a dissection microscope. The collected tissues were stored at -80 °C until the time of their use. Demarcation of the DCN, IC and AC tissues was guided by anatomical landmarks described in a pictorial atlas of the rat brain (Paxinos and Watson, 2007).

Brain extracts were prepared by dissolving (via trituration) microdissected tissue sections in 100μL of ice-cold lysis buffer (137mM NaCl, 5mM EDTA, 1% glycerol, 1% Triton X-100, 20mM Tris-HCl, pH 7.5), containing freshly-supplemented protease inhibitors. An aliquot from each of the primary tissue extracts was supplemented with SDS to a final concentration of 1%, and these aliquots were immediately heat denatured at 100°C. The protein concentration of each tissue extract was then determined with the Pierce BCA Protein Assay Kit, and equivalent amounts of total protein from each sample were supplemented with Laemmli buffer (2% SDS, 5% glycerol, 5% 2-mercaptoethanol, 0.002% bromphenol blue, 62.5mM Tris-HCl, pH 6.8) and resolved on 10% SDS-polyacrylamide gels. Resolved proteins were then electroblotted onto nitrocellulose membranes and blocked for 30 min. at room temperature with Tris-buffered saline solution containing 0.1% Tween 20 (TBST) and 3% dry milk. Immunoblotting was conducted in TBST using dilutions of the same GAP43 (1:2000) antibody used for immunohistology followed by incubation with appropriate HRP-conjugated secondary antibodies. The blots were washed extensively in TBST and visualized by chemiluminescence according to the manufacturer’s protocol (ECL Plus, GE Healthcare). Post-development, membranes were incubated at 50°C for 30 min. in stripping buffer (100mM 2-mercaptoethanol, 2% SDS, 62.5mM Tris-HCl, pH 6.7) to remove bound antibodies. The membranes were then washed in TBST, blocked with dry milk, and re-probed with an antibody against glyceraldehyde-3-phosphate dehydrogenase (GAPDH, 1:2000, Cell Signaling Technology), which served as an internal loading control.

Densitometric analyses were performed on digital scans of duplicate immunoblots for each target protein in a sample set, using the ImageJ processing software developed by the National Institutes of Health. The reported relative expression values for each target protein in a specific blast-exposed tissue sample represent an average of the normalized densities (relative to corresponding immunoblots for the internal GAPDH standards) from two to three independent evaluations relative to measured densities of the target protein in tissue samples dissected from the brains of naïve animals.
