# Supplementary figures and images for: Electrophysiological assessment and pharmacological treatment of blast-induced tinnitus
Source: PLoS One. 2021 Jan 7;16(1):e0243903. doi: 10.1371/journal.pone.0243903 (PMC7790300; doi:10.1371/journal.pone.0243903)

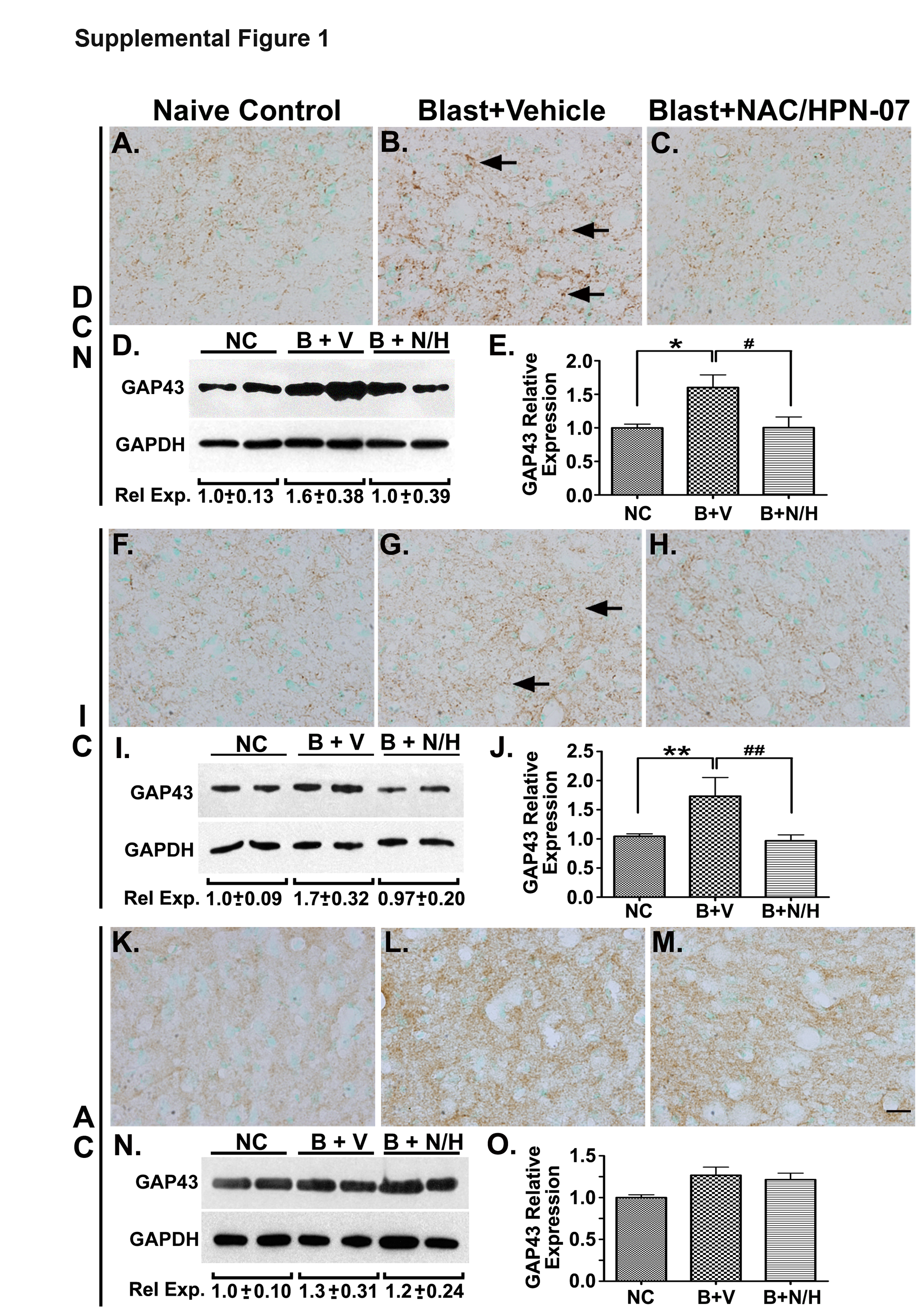

Supplement: S1 Fig — (TIF) [file pone.0243903.s002.tif]

DCN Raw Western Blots

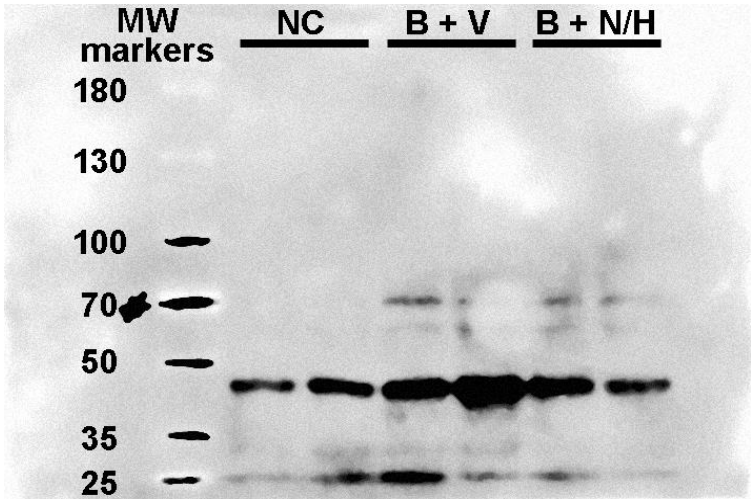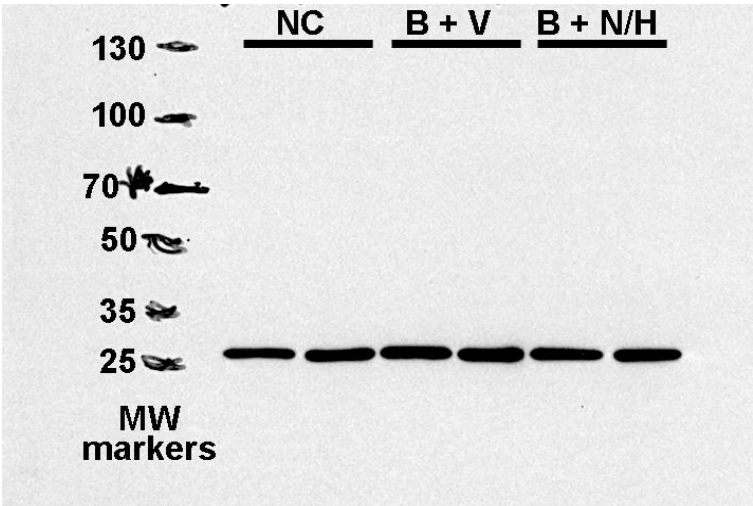

IC Raw Western Blots

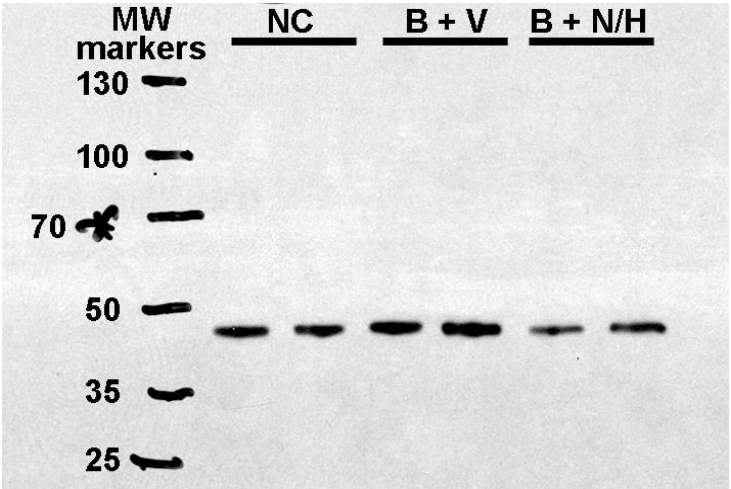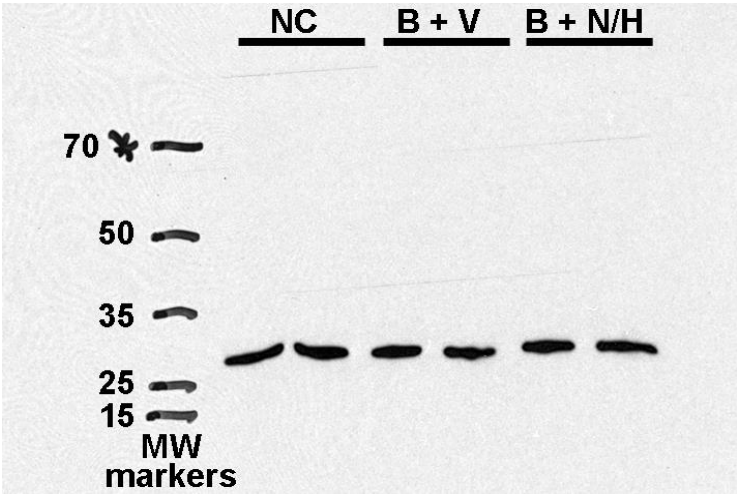

AC Raw Western Blots

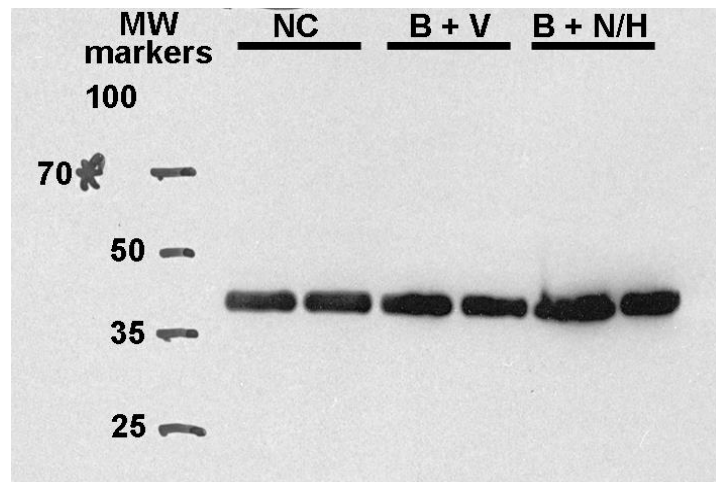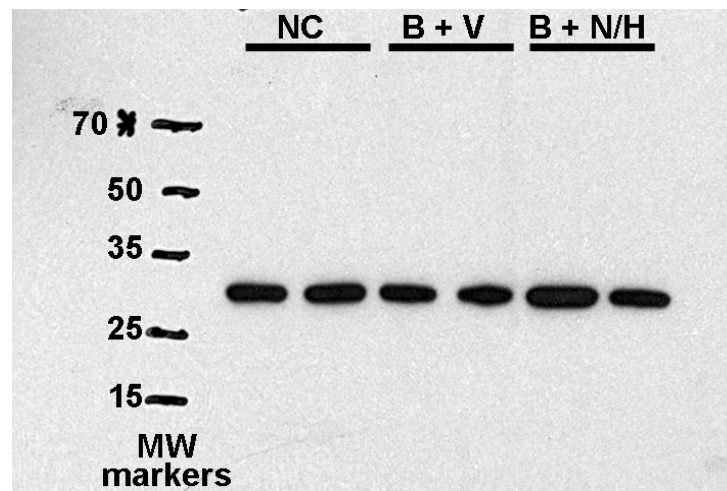

Supplement: S1 Raw Images — (PDF) [file pone.0243903.s003.pdf]
